# Machine-guided path sampling to discover mechanisms of molecular self-organization

**DOI:** 10.1038/s43588-023-00428-z

**Published:** 2023-04-24

**Authors:** Hendrik Jung, Roberto Covino, A. Arjun, Christian Leitold, Christoph Dellago, Peter G. Bolhuis, Gerhard Hummer

**Affiliations:** 1 Department of Theoretical Biophysics, Max Planck Institute of Biophysics, Frankfurt am Main, Germany; 2 Frankfurt Institute for Advanced Studies, Frankfurt am Main, Germany; 3 van ’t Hoff Institute for Molecular Sciences, University of Amsterdam, Amsterdam, The Netherlands; 4 Faculty of Physics, University of Vienna, Vienna, Austria; 5 Institute of Biophysics, Goethe University Frankfurt, Frankfurt am Main, Germany

**Keywords:** Chemical physics, Computational biophysics, Statistical physics

## Abstract

Molecular self-organization driven by concerted many-body interactions produces the ordered structures that define both inanimate and living matter. Here we present an autonomous path sampling algorithm that integrates deep learning and transition path theory to discover the mechanism of molecular self-organization phenomena. The algorithm uses the outcome of newly initiated trajectories to construct, validate and—if needed—update quantitative mechanistic models. Closing the learning cycle, the models guide the sampling to enhance the sampling of rare assembly events. Symbolic regression condenses the learned mechanism into a human-interpretable form in terms of relevant physical observables. Applied to ion association in solution, gas-hydrate crystal formation, polymer folding and membrane-protein assembly, we capture the many-body solvent motions governing the assembly process, identify the variables of classical nucleation theory, uncover the folding mechanism at different levels of resolution and reveal competing assembly pathways. The mechanistic descriptions are transferable across thermodynamic states and chemical space.

## Abstract (web summary)

A machine learning algorithm speeds up the sampling of rare assembly events, discovers their mechanisms, extrapolates them across chemical and thermodynamic space, and condenses the learned assembly mechanisms into a human-interpretable form.

## Main

Understanding how generic yet subtly orchestrated interactions cooperate in the formation of complex structures is the key to steering molecular self-assembly^1,2^. As computer experiments, molecular dynamics (MD) simulations promise us atomically detailed and unbiased views of self-organization processes^3^. However, most collective self-organization processes are rare events that occur on timescales many orders of magnitude longer than the fast molecular motions limiting the MD integration step. The system spends most of the time in metastable states, and the infrequent and rapid stochastic transitions between states are rarely resolved in unbiased MD simulations, if at all. These transition paths (TPs) are the very special trajectory segments that capture the reorganization process. Learning molecular mechanisms from simulations requires computational power to be focused on sampling TPs^4^ and distilling quantitative models from them^5^. Due to the high dimensionality of configuration space, both sampling and information extraction are exceedingly challenging in practice. Our algorithm addresses both challenges at once. It autonomously and simultaneously builds quantitative mechanistic models of complex molecular events, validates the models on the fly and uses them to accelerate the sampling by orders of magnitude compared with regular MD.

## Results

### Algorithm for physics-based mechanism learning

Statistical mechanics provides a general framework to obtain low-dimensional mechanistic models of self-organization events. In this Article, we focus on systems that reorganize between two states A and B (assembled or disassembled, respectively), but generalizing to an arbitrary number of states is straightforward. Each TP connecting the two states contains a sequence of snapshots capturing the system during its reorganization. Consequently, the transition path ensemble (TPE) is the mechanism at the highest resolution. As the transition is effectively stochastic, quantifying its mechanism requires a probabilistic framework. We define the committor *p*_*S*_(**x**) as the probability that a trajectory enters state *S* first, with *S* = A or B, respectively, where **x** is a vector of features representing the starting point **X** in configuration space, and *p*_A_(**x**) + *p*_B_(**x**) = 1 for ergodic dynamics. The committor *p*_B_ reports on the progress of the reaction A → B and predicts the trajectory fate in a Markovian way^6,7^, making it the ideal reaction coordinate^8,9^. In the game of chess, one can think of the committor as the probability of, say, black winning for given initial board positions in repeated games^10^. The minimal requirements for applications beyond molecular simulations are (1) that a quantity akin to a committor exists and (2) that the dynamics of the system can be sampled repeatedly, at least in the forward direction. The probability of different possible events (A, B, …) should thus be encoded at least in part (and thus learnable in terms of) the instantaneous state **X** of the system and the dynamics of the system should be amenable to repeated sampling, preferably by efficient computer simulation. However, if one can repeatedly prepare a real system with satisfactory control over the initial conditions, one can learn to predict the likely fate of this system given the observed and controlled initial conditions using our framework.

Sampling TPs for rare events and learning the associated committor function *p*_B_(**x**) are two outstanding and intrinsically connected challenges. Given that TPs are exceedingly rare in a very high-dimensional space, an uninformed search is futile. However, TPs converge near transition states^7^, where the trajectory is least committed with *p*_A_(**x**) = *p*_B_(**x**) = 1/2. For Markovian and time-reversible dynamics *P*(TP|**x**), the probability for a trajectory passing through **x** to be a TP, satisfies *P*(TP|**x**) = 2*p*_B_(**x**)(1 − *p*_B_(**x**)), that is, the committor determines the probability of sampling a TP^11^. The challenges of information extraction and sampling are thus intertwined.

**Fig. 1:**
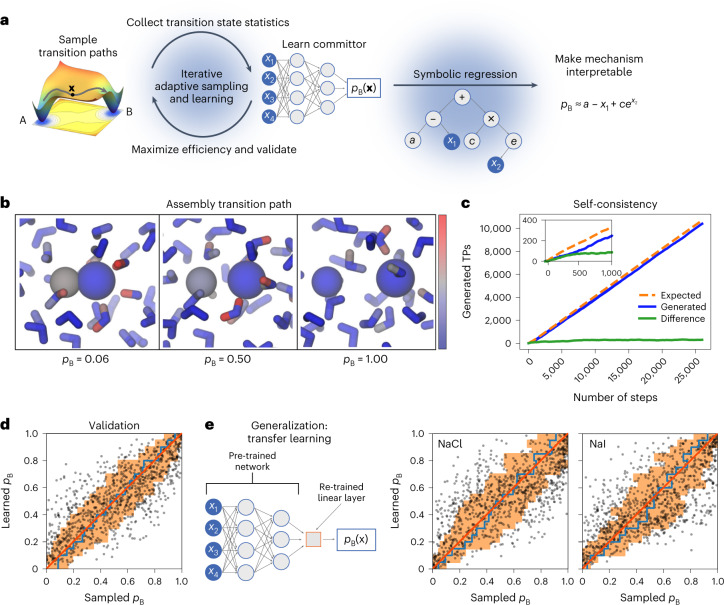
Learning the assembly mechanism of ions in water. **a**, Mechanism learning by path sampling. The method iterates between sampling transition paths from a configuration **x** between metastable states A and B (left), and learning the committor *p*_B_(**x**) (right). A neural network function of molecular features (*x*_1_ to *x*_4_) models the committor. The log predictor forming the last layer is not shown. At convergence, symbolic regression distills an interpretable expression that quantifies the molecular mechanism in terms of selected features (*x*_1_, *x*_2_) and numerical constants (*a*, *c*) connected by mathematical operations (here: +, −, ×, exp). **b**, Snapshots along a TP showing the formation of a LiCl ion pair (right to left) in an atomistic MD simulation. Water is shown as sticks, Li^+^ as a small sphere and Cl^−^ as a large sphere. Atoms are colored according to their contribution to the reaction progress from low (blue) to high (red), as quantified by their contribution to the gradient of the reaction coordinate *q*(**x**|**w**). **c**, Self-consistency. Counts of the generated (blue line) and expected (orange dashed line) number of transition events. The green line shows the cumulative difference between the observed and expected counts. The inset shows a zoom-in on the first 1,000 iterations. **d**, Validation of the learned committor. Cross-correlation between the committor predicted by the trained network and the committor obtained by repeated sampling from molecular configurations on which the committor model was not trained. The average of the sampled committors (blue line) and their s.d. (orange shaded) were calculated in bins of the learned committor indicated by the vertical steps. For reference, the red line indicates the identity. **e**, Transferability of the learned committor. Representation of transfer learning, and cross-correlations between sampled committors for NaCl and NaI ion pairing and predictions of committor from a model trained on data for LiCl and adjusted by transfer learning using only 1,000 additional shooting outcomes each. Colors and s.d. (indicated by orange shading) are as in **d**. Source data

To tackle these dual challenges, we designed an iterative algorithm that builds on transition path theory^9^ and transition path sampling (TPS)^12^ in the spirit of reinforcement learning. The algorithm learns the committor of rare events in complex many-body systems by repeatedly running virtual experiments and uses the knowledge gain to improve the sampling of TPs (Fig. 1a). In each experiment, the algorithm selects a point **X** from which to shoot trajectories—propagated according to the unbiased dynamics of the physical model—to generate TPs. To ensure detailed balance of TPS, the algorithm selects structures from the current transition path to initiate shooting moves with redrawn Maxwell–Boltzmann initial velocities. After repeated shots from different points **X**, the algorithm compares the number of generated TPs with the expected number based on its knowledge of the committor at that point. Only if the prediction is poor, the algorithm retrains the model on the outcome of all virtual experiments, which prevents overfitting. As the predictive power of the mechanistic model increases, the algorithm becomes more efficient at sampling TPs by choosing initial points near transition states, that is, according to *P*(TP|**x**).

The algorithm learns from its repeated attempts by using deep learning^5,13,14^ in a self-consistent way. We model the committor *p*_B_(**x**) = 1/(1 + e^−*q*(**x**|**w**)^) with a neural network^15^ *q*(**x**|**w**) of weights **w**. With this choice, *q* is an invertible function of *p*_B_, and we can view both functions as the reaction coordinate. In each attempt to generate a TP, the algorithm propagates two trajectories, one forward and one backward in time, by running MD simulations^4^. In case of success, one trajectory first enters state A and the other B, jointly forming a new TP. In this Bernoulli coin-toss process, the algorithm learns from both successes and failures. The negative log-likelihood^5^ for *k* attempts defines the loss function $l({{{\bf{w}}}}| {{{\bf{\uptheta }}}})=\mathop{\sum }\nolimits_{i = 1}^{k}\log (1+{{\mathrm{e}}}^{{s}_{i}q({{{{\bf{x}}}}}_{i}| {{{\bf{w}}}})})$, where *s*_*i*_ = 1 if trajectory *i* initiated from **X**_*i*_ enters A first, and *s*_*i*_ = −1 if it enters B first. The training set **θ** contains the *k* feature vectors **x**_*i*_ associated with the shooting points **X**_*i*_ and outcomes *s*_*i*_. By training the network *q*(**x**|**w**) to minimize the loss *l*(**w**|**θ**), we obtain a maximum likelihood estimate of the committor^5^ that is differentiable and enables sophisticated analysis methods^16^.

We then use symbolic regression^17^ to condense the molecular mechanism into a human-interpretable form (Fig. 1a) and gain physical insight. First, an attribution analysis of the trained network identifies a small subset **z** of the input coordinates **x** that dominate the outcome of the network prediction. Then, symbolic regression distills explicit mathematical expressions *q*_sr_(**z**|**w**_sr_) by using a genetic algorithm that searches both function and parameter spaces to minimize the loss *l*(**w**_sr_|**θ**) on the training set **θ** independent of the preceding neural network training, where the subscript ‘sr’ indicates symbolic regression. The resulting analytical expressions provide us with a list of hypotheses for quantitative models of the physics governing the molecular assembly process. For further examination, these hypotheses are ranked by a combination of statistical likelihood (that is, how well they account for all available data) and mathematical complexity.

### Ion assembly in solution

The formation of ion pairs in water is a paradigmatic assembly process controlled by many-body interactions in the surrounding solvent medium. Even though MD can efficiently simulate the process, the collective reorganization of water molecules challenges the formulation of quantitative mechanistic models to this day^18^ (Fig. 1b).

**Extended Data Fig. 1:**
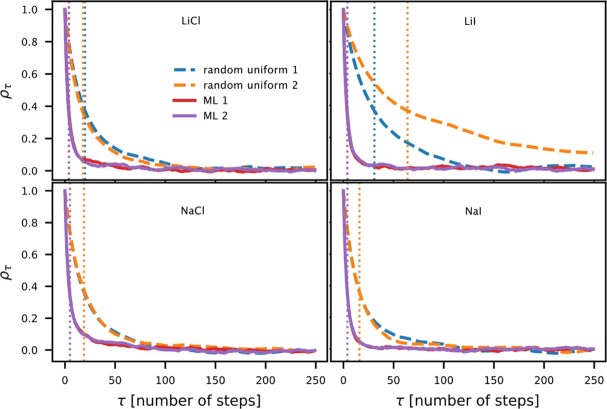
Sampling efficiency compared to random selection two-way shooting TPS. Normalized autocorrelation function of the transition time *t*_*TP*_ as a function of the number of Monte Carlo (MC) steps for TPS simulations on different ionic species, in which the shooting points were selected by the algorithm (red and green curves, independent runs) or by random uniform selection (orange and blue curve, independent runs). Each autocorrelation function was calculated over MC chains with a length of 100000 steps each. The decorrelation times of the Markov chains, defined as the points where the normalized autocorrelation functions reach the value *e*^−1^, are marked with dotted vertical lines in the same color as the respective autocorrelation function. Machine-guided sampling leads to decorrelation times of four MC steps in all cases, while for random uniform selection this value ranges between 20-60 steps depending on the ionic species. Source data

The algorithm quickly learned how to optimally sample the formation of ion pairs. For lithium (Li^+^) and chloride (Cl^−^) ion pairs in water (Fig. 1b,c), the network used the interionic distance *r*_LiCl_ and 220 molecular features *x*_1_, …, *x*_220_ that describe the angular arrangement of water oxygen and hydrogen atoms at a specific distance from each ion^19^. These coordinates provide a general representation of the system that is invariant with respect to physical symmetries and exchange of atom labels. After the first 500 iterations, the predicted and observed numbers of TPs agree (Fig. 1c). Sampling is about ten times faster than conventional TPS (Extended Data Fig. 1). We note that this speed-up is achieved entirely by improving the efficiency of sampling new transition paths and without bias on the underlying dynamics. We further validated the learned committor function by checking its predictions against independent simulations. From 1,000 configurations not used in training, we initiated 500 independent simulations each and estimated the sampled committor *p*_B_ as the fraction of trajectories first entering the unbound state. Predicted and sampled committors are in quantitative agreement (Fig. 1d).

**Extended Data Fig. 2:**
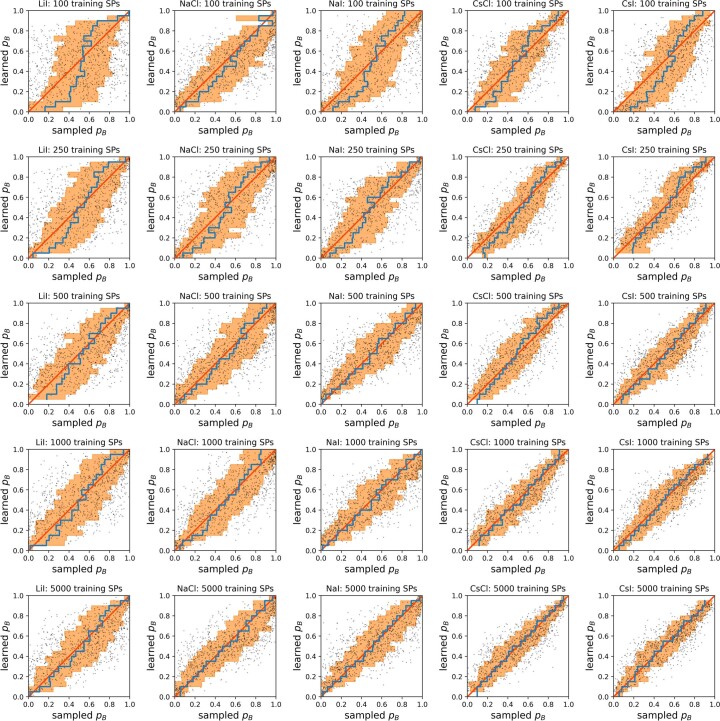
Transfer learning of ion association in solution. Committor cross-correlation plots for models obtained by transfer training on different ionic species with varying number of training shooting points. The original model was trained on LiCl and is the same in all cases (see Fig. 1 for a committor cross-correlation plot of the original model predicting for LiCl). The model is of architecture ‘ResNet I’ and uses the inputs ‘SF longranged II’ (see Supplementary Tables 5 and 6). Transfer training was done by randomizing the last layer of the original model with a single neuron and then training on the specified number of shooting points with data for each ionic system separately. The average of the sampled committors (blue line) +/- one SD (orange shaded) were calculated by binning the sampled committor in the range of the learned committor indicated by the vertical steps. For reference, the red line indicates the identity. Source data

Counter to a common concern for machine learning models, the learned mechanism is general and, with minimal adjustments, describes the assembly of chemically distinct ionic species. We performed transfer learning on five additional systems by allowing modifications in only the last linear layer of the trained network containing a single neuron (Fig. 1e and Extended Data Fig. 2). A very limited amount of new simulated transitions is sufficient to adjust the network containing the LiCl committor to correctly predict the committor for LiI, NaCl, NaI, CsCl and CsI.

**Extended Data Fig. 3:**
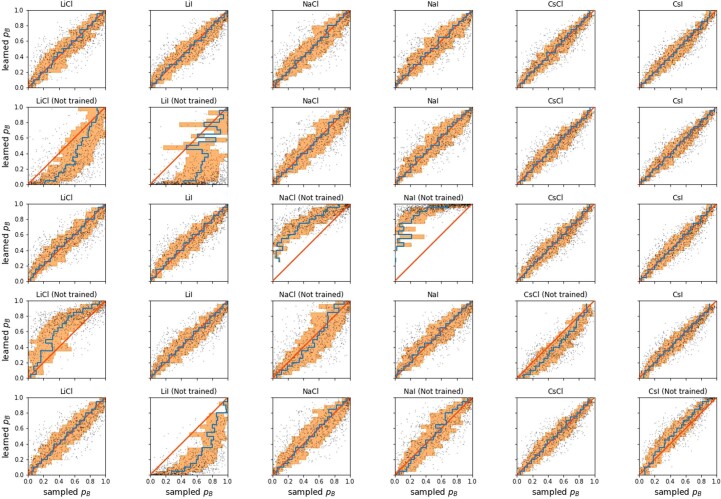
Committor validation of a multi-ion assembly models and extrapolation in chemical space. Shown are cross-correlations of sampled committors (*x* axis) and predicted committors (*y* axis) for models trained on different subsets of the six different ion pairs. (Row 1) Training on all six ion pairs (Li^+^ Cl^−^, Li^+^ I^−^, Na^+^ Cl^−^, Na^+^ I^−^, Cs^+^ Cl^−^, and Cs^+^ I^−^) simultaneously. (Row 2) Model trained without data for Li^+^, that is, excluding Li^+^ Cl^−^ and Li^+^ I^−^. (Row 3) Model trained without data for Na^+^, that is, excluding Na^+^ Cl^−^ and Na^+^ I^−^. (Row 4) Model trained without data for Cl^−^, that is, excluding Li^+^ Cl^−^, Na^+^ Cl^−^, and Cs^+^ Cl^−^. (Row 5) Model trained without data for I^−^, that is, excluding Li^+^ I^−^, Na^+^ I^−^, and Cs^+^ I^−^. All models are of architecture ‘ResNet I’ (see Supplementary Table 6). For all models, the different ionic species were distinguished by adding the *ϵ* and *σ* Lennard-Jones parameters of the force field as additional descriptors to the set ‘SF shortranged’ (see Supplementary Table 5), which was used to describe the solvent around the ion pair. The average of the sampled committors (blue line) +/- one SD (orange shaded) were calculated by binning the sampled committor in the range of the learned committor indicated by the vertical steps. For reference, the red line indicates the identity. Source data

We also built multi-ion models extending across chemical space. As reporters on ion size and energetics, we included the parameters particle size *σ* and dispersion energy *ϵ* of the Lennard-Jones potential in the feature vectors **x**. We found that models trained on the combined TP statistics for different ion-pair combinations can inter- and extrapolate in chemical space, making reasonable predictions for the association mechanism of ion species it has not trained on (Extended Data Fig. 3).

### Interpretable mechanism across chemical space

**Fig. 2:**
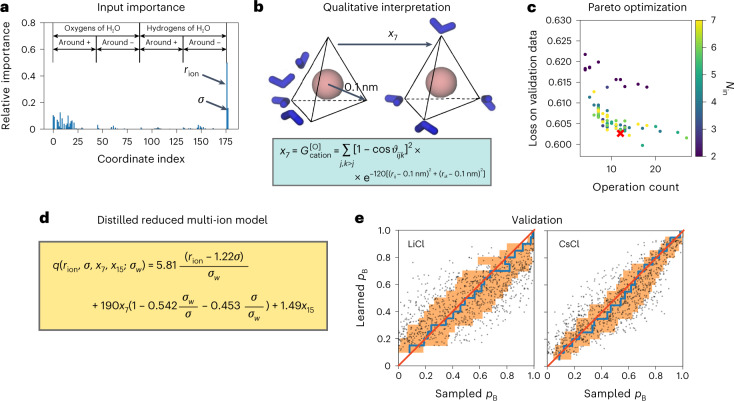
Interpretable multi-ion model of the assembly mechanism of ions in water. **a**, Input relevance for all 179 input coordinates used for deep learning. The first 176 describe the geometry of water molecules around cations and anions. The remaining ones are the interionic distance *r*_ion_ and the Lennard-Jones parameters, with *σ* the ion size. **b**, Schematic depiction of the most important solvent reorientation. The symmetry function *x*_7_ reports the water oxygen atoms (O, in blue) geometry at 0.1 nm around the cation (in pink) (see the box for the definition of *x*_7_, where *r*_*ij*_ and *r*_*ik*_ are the distances between the central cation *i* and oxygen atoms *j* and *k*, and *ϑ*_*ijk*_ is the angle formed by the central cation and two oxygen atoms). **c**, Pareto plot of all models distilled by symbolic regression. Each dot corresponds to an alternative model *q*_sr_(**z**|**w**_sr_), colored according to the number of input coordinates (*N*_in_) it uses. The red cross identifies the optimal model at the knee of the Pareto front. **d**, Multi-ion model from symbolic regression describing the assembly mechanism of LiCl, LiI, NaCl, NaI, CsCl and CsI in water. The model, $q\left({r}_{{\mathrm{ion}}},\sigma ,{x}_{7},{x}_{15};{\sigma }_{w}\right)$, is a function of the interionic distance (*r*_ion_ and ion size *σ* in units of the water size parameter *σ*_*w*_ = 0.315 nm) and the geometry of water around the cations (*x*_7_ and *x*_15_). **e**, Validation of the multi-ion assembly model by cross-correlation between untrained sampled committors and the prediction for each ionic species separately, here shown for LiCl and CsCl (see Extended Data Fig. 4a for all remaining species). The average of the sampled committors (blue line) and their s.d. (orange shaded) were calculated in bins of the learned committor indicated by the vertical steps. For reference, the red line indicates the identity. Source data

Solvent rearrangements play a critical role in determining ion assembly. Attribution analysis for a model trained on LiCl, LiI, NaCl, NaI, CsCl and CsI assembly simultaneously identified the interionic distance *r*_ion_ and the Lennard-Jones parameters as crucial (Fig. 2a). In addition, the symmetry functions describing the geometry of water molecules around the cation control the assembly mechanism. As the most important of the 176 molecular features used to describe the solvent, *x*_7_ quantifies oxygen anisotropy at a radial distance of 0.1 nm from the cations (Fig. 2b). For successful ion-pair assembly, these inner-shell water molecules need to open up space for the incoming anion. The importance of inner-shell water rearrangement is consistent with a visual analysis that highlights atoms in a TP according to their contribution to the committor gradient (Fig. 1b).

Symbolic regression provides a quantitative and interpretable multi-ion model of the assembly mechanism. In independent symbolic regressions, we varied the number of inputs and the complexity penalty. We then selected models in a Pareto plot (Fig. 2c). Models at the knee of the Pareto front offer good trade-offs between model quality, as measured by the loss, and model complexity, as measured by the number of mathematical operations.

**Extended Data Fig. 4:**
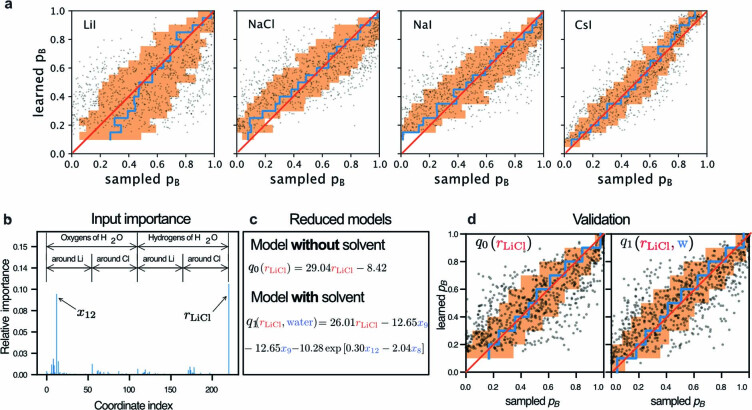
Reduced interpretable models of ion assembly. **a**, Validation of multi-ion reduced model for additional four ion pairs. The average of the sampled committors (blue line) and their standard deviation (orange shaded) are calculated by binning along the predicted committor (red line: identity). **b**, Most important input coordinates determining the committor trained on association simulations of a single ionic pair, in this case LiCl (see also Supplementary Table 13 for a listing of the ten most relevant coordinates). **c**, Reduced models *q*_0_ and *q*_1_ describing association of Li^+^ Cl^−^ in water obtained by symbolic regression at strict (*λ* = 10^−6^) and gentle regularization (*λ* = 10^−7^), respectively. Note that the first model does not depend on water degrees of freedom (see again Supplementary Table 13 for a description of the coordinates). **d**, Cross-correlation plots between untrained committor data and the symbolic regression predictions as independent validations of the accuracy of *q*_0_ and *q*_1_. The average of the sampled committors (blue line) +/- one SD (orange shaded) are calculated by binning along the predicted committor (red line: identity). Source data

The distilled multi-ion model is interpretable and provides physical insight into the assembly of monovalent ions in water (Fig. 2d). In the leading term in *q*, a scaled ion-size parameter *σ* is subtracted from the interionic distance, consistent with physical intuition. In the second term, ion size nonlinearly modulates the descriptor *x*_7_ of water geometry close to the cations (Fig. 2b). In the last term, *x*_15_ reports on solvation farther away unmodulated by ion identity. Despite its simplicity, the reduced model is accurate for all monovalent ion species considered here (Fig. 2e and Extended Data Fig. 4a). A symbolic regression model focusing on the assembly of LiCl only shows that we can trade less generality for higher accuracy (Extended Data Fig. 4b–d).

### Gas-hydrate crystal formation

**Fig. 3:**
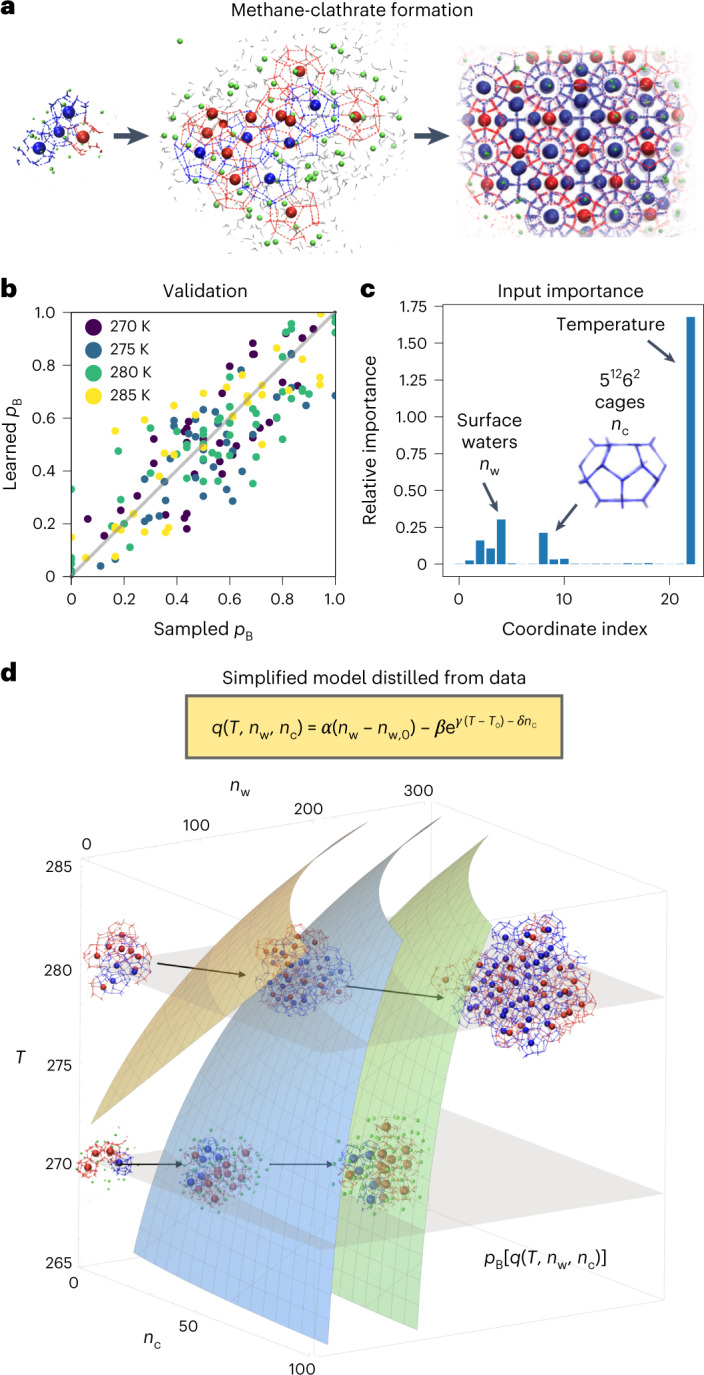
Data-driven discovery of methane-clathrate nucleation mechanism. **a**, Molecular configurations illustrating the nucleation process extracted from an atomistic MD simulation in explicit solvent. The nucleus forms in water, grows and leads to the clathrate crystal. 5^12^6^2^ (blue) and 5^12^ (red) water cages (lines) contain correspondingly colored methane molecules (spheres). Methane molecules near the growing solid nucleus are shown as green spheres and water as gray sticks. Bulk water is not shown for clarity. **b**, Validation of the learned committor. Cross-correlation between the committor predicted by the trained network and the committor obtained by repeated sampling from molecular configurations on which the algorithm did not train (gray line: identity). **c**, Input importance analysis. The three most important input coordinates are annotated as temperature *T*, the number of surface waters *n*_w_ and the number of 5^12^6^2^ crystals *n*_c_. **d**, Data-driven quantitative mechanistic model distilled by symbolic regression reveals a switch in nucleation mechanism. In the equation, *n*_w,0_ and *T*_0_ are the reference number of surface water molecules and the reference temperature, respectively, and *α*, *β*, *γ* and *δ* are numerical constants. Analytical iso-committor surfaces for *n*_w,0_ = 2, *T*_0_ = 270 K, *α* = 0.0502, *β* = 3.17, *γ* = 0.109 K^−1^, *δ* = 0.0149 (left to right: yellow, *p*_B_ = 1/(1 + e^−4^); blue, 1/2; green, 1/(1 + e^4^)). The structural insets illustrate the two competing mechanisms at low and high temperature. Source data

At low temperature and high pressure, a liquid mixture of water and methane organizes into a gas hydrate, an ice-like solid^20^. In this phase transition, water molecules assemble into an intricate crystal lattice with regularly spaced cages filled by methane (Fig. 3a). Despite commercial relevance in natural gas processing, the mechanism of gas-hydrate formation remains incompletely understood, complicated by the many-body character of the nucleation process and the competition between different crystal forms^20^. Studying the nucleation mechanism is challenging for experiments and, due to the exceeding rarity of the events, impossible in equilibrium MD.

**Extended Data Fig. 5:**
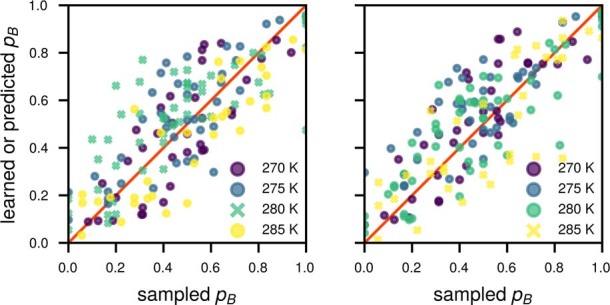
Interpolation and extrapolation in temperature for methane clathrate nucleation models. Cross-correlation between learned committor and the committor as obtained by repeated sampling on untrained configurations for two models which are trained on only three of the four temperatures available in the training set. (Left) Committor model trained only on data for *T*=270 K, 275 K, and 285 K to assess the ability of the model to interpolate to *T*=280 K. (Right) Model trained on data for T=270 K, 275 K, and 280 K to assess the ability of the model to extrapolate to *T*=285 K. The red line represents the identity. Source data

Within hours of computing time on a single graphics processing unit (GPU), the algorithm extracted the nucleation mechanism from 2,225 TPs showing the formation of methane clathrates, corresponding to a total of 445.3 μs of simulated dynamics. The trajectories were produced by extensive TPS simulations at four different temperatures, and provided a pre-existing training set for our algorithm^21^. We described molecular configurations by using 22 features commonly used to describe nucleation processes (Supplementary Table 1). We considered the temperature *T* at which a TP was generated as an additional feature, and trained the committor model on the cumulative trajectories. We showed that the learned committor as a function of temperature is accurate by validating its predictions for 160 independent configurations (Fig. 3b). Generative models recently constructed distribution functions at temperatures not sampled^22^. By leaving out data at *T* = 280 K or 285 K in the training, we show that the learned committor satisfactorily interpolates and extrapolates to thermodynamic states not sampled (Extended Data Fig. 5).

Temperature *T* is the most critical factor for the outcome of a simulation trajectory, followed by the number *n*_w_ of surface water molecules and the number *n*_c_ of 5^12^6^2^ cages, defined by the presence of 12 pentagons (5^12^) and two hexagons (6^2^) (Fig. 3c). All three variables play an essential role in the classical theory of homogeneous nucleation^21^. The activation free energy Δ*G* for nucleation is determined by the size of the growing nucleus, parameterized by the amount of surface water and—in case of a crystalline structure—the number of 5^12^6^2^ cages. Temperature determines, through the degree of supersaturation, the size of the critical nucleus, the nucleation free energy barrier height and the rate.

Symbolic regression distilled a mathematical expression revealing a temperature-dependent switch in the nucleation mechanism. The mechanism is quantified by *q*(*n*_w_, *n*_c_, *T*) (Fig. 3d and Supplementary Table 1). At low temperatures, the size of the nucleus alone decides on growth. At higher temperatures, the number of 5^12^6^2^ water cages gains in importance, as indicated by curved iso-committor surfaces (Fig. 3d). This mechanistic model, generated in a data-driven way, reveals the switch from amorphous growth at low temperatures to crystalline growth at higher temperatures^21,23^.

### Polymer folding at different resolutions

**Fig. 4:**
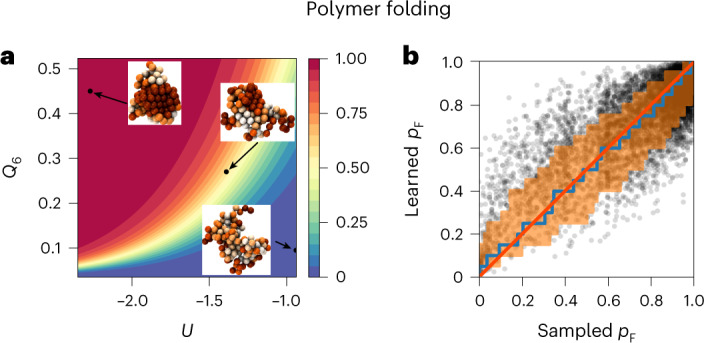
Learning the mechanism of polymer folding. **a**, Representation of the learned mechanism. The heat map (color bar) represents a reduced explicit model of the committor *p*_B_ = *p*_F_ to the folded state as reproduced by the expression ${q}_{{\mathrm{B}}}\left(U,{Q}_{6}\right)=\alpha (U-{U}_{0})+\beta \log \left({Q}_{6}-{Q}_{6,0}\right)+\gamma$, where *U* is the total potential energy of the polymer, *Q*_6_ quantifies its crystallinity, and the numerical constants are *α* = −7.144, *β* = 3.269, *γ* = 11.942, *U*_0_ = −2.351 and *Q*_6,0_ = 0.035. Insets: molecular configurations of the polymer at *p*_B_ = 0, 0.5 and 1. Polymer beads are colored according to their value of *q*_6_, from white (low values) to dark orange (high values). **b**, Validation of the learned committor. Cross-correlation between the committor predicted by the trained network and the committor obtained by repeated sampling from molecular configurations on which the algorithm did not train. The average of the sampled committors (blue line) and their s.d. (orange shaded) were calculated in bins of the learned committor indicated by the vertical steps. For reference, the red line indicates the identity. Source data

**Extended Data Fig. 6:**
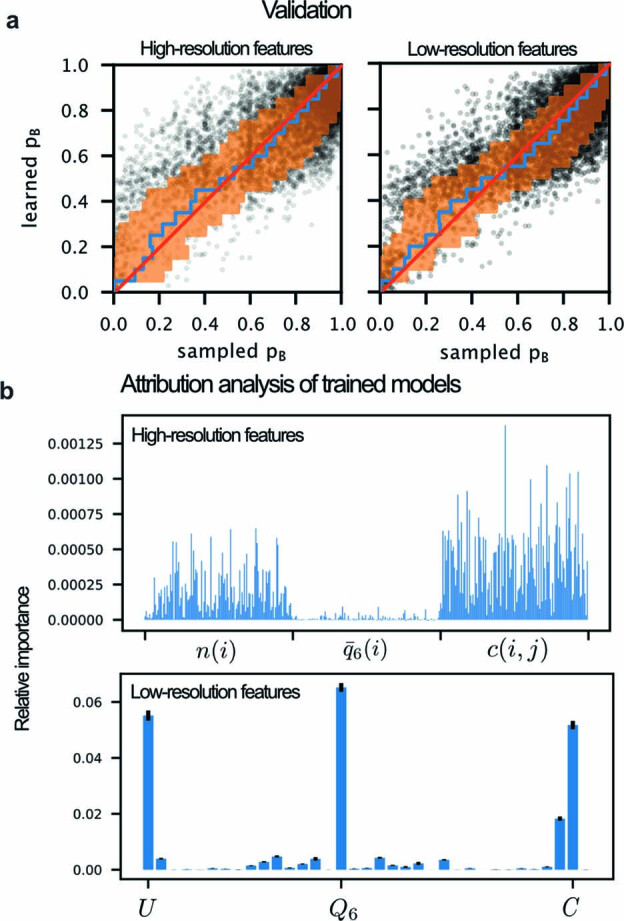
Committor of polymer folding as a function of high- and low-resolution features. **a**, Committor cross correlation on untrained configurations. **b**, Input relevance for neural networks trained on polymer nucleation data using low-resolution and high-resolution features, respectively. In **a**, the average of the sampled committors (blue line) and their standard deviation (orange shaded) were calculated by binning along the predicted committor (red line: identity). In **b**, the blue bars show the mean of *n* independent repetitions of the input importance analysis (*n* = 100 for high resolution features and n=250 for low resolution features), and the error bars indicate +/- one SD. Source data

Proteins, nucleic acids and polymers can spontaneously self-organize by folding into ordered structures. Applied to the coil-to-crystal transition of a homopolymer^24,25^, the algorithm readily identified the previously elusive mechanism at different levels of resolution (Extended Data Fig. 6). At low resolution, we used a select set of 36 physical characteristics averaged over the polymer. Attribution analysis followed by symbolic regression represented the committor as a nonlinear function of orientational order *Q*_6_ and potential energy *U* alone, which proved highly predictive (Fig. 4 and Extended Data Fig. 6a). At high resolution, deep learning produced a committor function of comparable quality in a space of 384 general descriptors representing the local environment of each polymer bead (Extended Data Fig. 6b) in terms of the number of neighbors, the local bond-order parameter *q*_6_ and the connection coefficients *c*_*ij*_ that measure the correlation between the local environments of beads *i* and *j*. The algorithm thus learned accurate committor representations in terms of both many general and few system-specific features, and distilled the latter into a compact and physically insightful function of orientational order and energy.

### Competing pathways for membrane-protein complex assembly

**Fig. 5:**
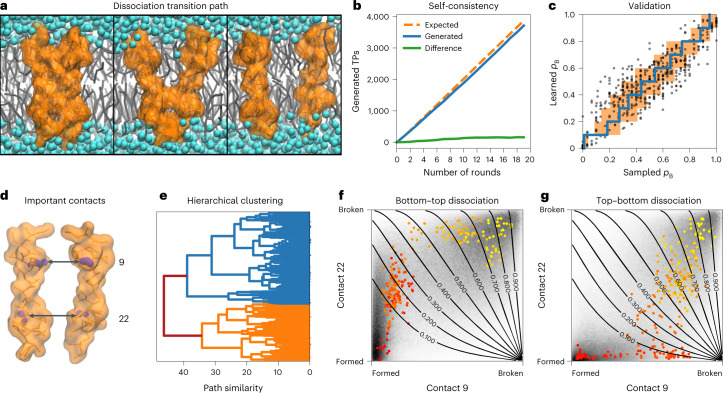
Competing pathways of transmembrane dimer assembly in lipid membrane. **a**, Snapshots during a Mga2 dimerization event (right to left). The transmembrane helices are shown as orange surfaces, the lipid molecules in gray and water in cyan. **b**, Self-consistency. Cumulative counts of the generated (blue line) and expected (orange dashed line) number of transitions. The green curve shows the cumulative difference between the observed and expected counts. **c**, Validation of the learned committor. Cross-correlation between the committor predicted by the trained network and the committor obtained by repeated sampling from molecular configurations on which the committor model was not trained. The average of the sampled committors (blue line) and their s.d. (orange shaded) were calculated in bins of the learned committor indicated by the vertical steps. For reference, the red line indicates the identity. **d**, Schematic representation of the two most relevant coordinates, the interhelical contacts at positions 9 and 22. **e**, Hierarchical clustering of all TPs. Dendrogram as a function of TP similarity (dynamic time warping, see ‘Mga2 transmembrane dimer assembly in lipid membrane’ in Methods) calculated in the plane defined by contacts 9 and 22 (two main clusters: blue, orange). **f**,**g**, Path density (gray shading) for the two main clusters in **e**, calculated in the plane defined by contacts 9 and 22. For each cluster, one representative TP is shown from unbound (yellow) to bound (red). The isolines of the committor, as predicted by the symbolic regression ${q}_{{\mathrm{B}}}({x}_{9},{x}_{22})=-\exp ({x}_{9}^{2})\log ({x}_{9}-\frac{{x}_{9}}{\log ({x}_{22})})$, are shown as labeled solid lines. Source data

Membrane-protein complexes play a fundamental role in the organization of living cells. Here we investigated the assembly of the transmembrane homodimer of the saturation sensor Mga2 in a lipid bilayer in the quasi-atomistic Martini representation (Fig. 5a)^26^. In extensive equilibrium MD simulations, the spontaneous association of two Mga2 transmembrane helices has been observed, yet no dissociation occurred in approximately 3.6 ms (equivalent to more than 6 months of calculations)^26^.

Path sampling is naturally parallelizable, which enabled us to sample nearly 4,000 dissociation events in 20 days on a parallel supercomputer (Fig. 5b). The time integration of MD trajectories incurs the highest computational cost and is only parallelizable to a limited degree. However, a single instance of the algorithm can simultaneously orchestrate virtual experiments on an arbitrary number of copies of the physical model (by guiding parallel Markov chain Monte Carlo (MC) sampling processes), and learn from all of them by training on the cumulative outcomes.

**Extended Data Fig. 7:**
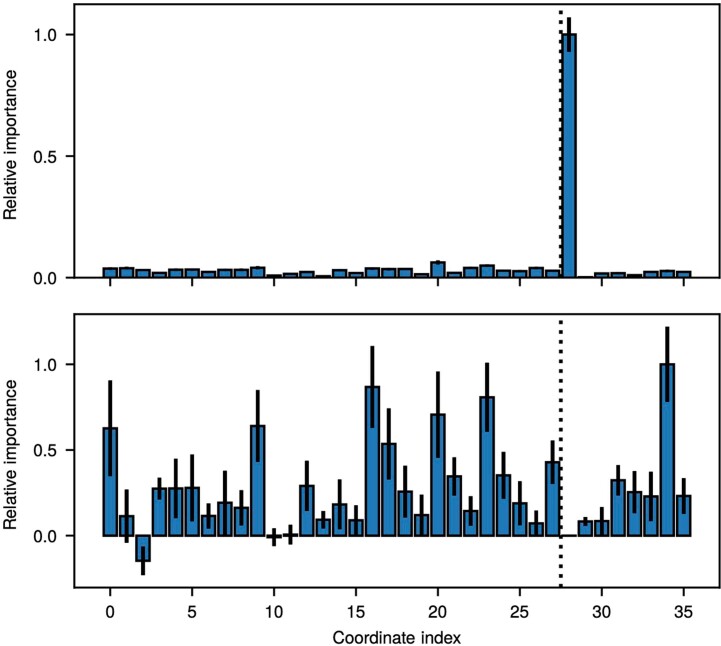
Input importance analyses for Mga2 transmembrane assembly. Input importance analyses by using all training points (top panel), and a subset with *n*_contacts_ < 0.01 (bottom panel), corresponding to training points close to the unbound state. The height of each bar is the mean over 50 independent analyses (*n* = 50), while the bars indicate +/- one SD. All values are normalized to the largest importance in each set. Source data

We featurized molecular configurations using contacts between corresponding residues along the two helices and included, for reference, a number of hand-tailored features describing the organization of lipids around the proteins^27^ (Extended Data Fig. 7 and Supplementary Table 2). We validated the model against committor data for 548 molecular configurations not used in training, and found the predictions to be accurate across the entire transition region between bound and unbound states (Fig. 5c).

**Extended Data Fig. 8:**
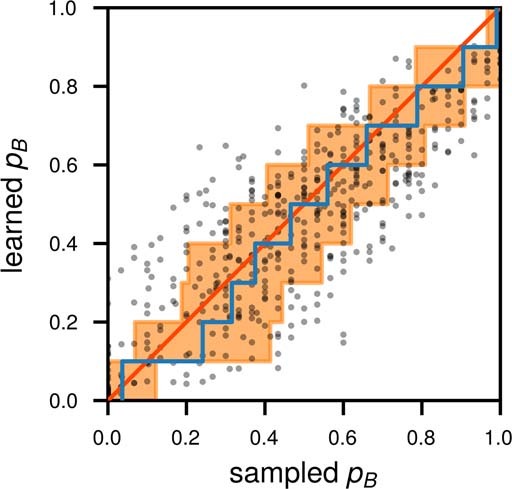
Committor cross correlation plot for symbolic regression expression for Mga2 assembly. Committor cross correlation plot for symbolic regression expression ${q}_{B}({x}_{9},{x}_{2}2)=-\exp ({x}_{9}^{2})\log ({x}_{9}-\frac{{x}_{9}}{\log ({x}_{22})})$ on untrained validation committor data of Mga2 transmembrane assembly. The expression is a function of interhelical contact 9 (*x*_9_) at the top of the two helices and contact 22 (*x*_22_) at the bottom (see Supplementary Tables 2 and 3). The average of the sampled committors (blue line) and their standard deviation (orange shaded) are calculated by binning along the predicted committor (red line: identity). Source data

In a remarkable reduction of dimensionality, symbolic regression achieved an accurate representation of the learned committor as a simple function of just two amino acid contacts (Fig. 5d and Extended Data Fig. 8). Symbolic regression provides us with a list of hypotheses for quantitative models of the physics governing the molecular assembly process (Supplementary Table 3). These hypotheses are ranked by a combination of statistical likelihood (that is, how well they account for all available data) and their mathematical complexity. Among the expressions at the knee of the Pareto plot, that is, with comparable predictive power and complexity, we chose the one offering a clear interpretation in terms of two competing assembly mechanisms described by the formation of contacts starting on one helix terminus or the other.

We projected all sampled TPs on the plane defined by these two contacts, calculated the distances between them and performed a hierarchical trajectory clustering (Fig. 5e). TPs organize in two main clusters that reveal two competing assembly pathways with the initial helix contact at the top (Fig. 5f) or the bottom (Fig. 5g). Unexpectedly^27^, helix–dimer geometry alone predicts assembly progress, which implies that the lipid ‘solvent’ is implicitly encoded in the interhelical pairwise contacts, unlike the water solvent in ion-pair formation^18^. As in polymer folding and ion binding, a sufficiently large space of general geometric features thus proved sufficient for the construction of fully predictive committors by deep learning. This finding is consistent with embedding theory^28^ and implies that the use of a small but sufficient number of general features is as effective as collective variables based on physical and chemical intuition.

## Discussion

Machine-guided trajectory sampling is general and can immediately be adapted to sample many-body dynamics with a notion of ‘likely fate’ similar to the committor. This fundamental concept of statistical mechanics extends from the game of chess^10^ over protein folding^3,7^ to climate modeling^29^. The simulation engine—MD in our case—is treated as a black box and can be replaced by other dynamic processes, reversible or not. Both the statistical model defining the loss function and the machine learning technology can be tailored for specific problems. More sophisticated models will be able to learn more from less data or incorporate experimental constraints. Simpler regression schemes^5^ can replace neural networks^15^ when the cost of sampling trajectories severely limits the volume of training data.

Defining the boundaries of the metastable states, as required by our method, can be non-trivial. To refine the state definitions, the committor framework can be used in an iterative way, starting from a very conservative one, obtaining a rough estimate of the committor, and then extending the states to values of the committor close to 0 and 1. A related problem is the presence of unassigned metastable states that result in long transition paths. Here, machine learning approaches aimed at state discovery may provide a solution.

As our method does not use un-physical forces to speed up the sampling, its applicability is limited to processes for which the transition paths are short enough so that the underlying simulation engine can sample a reasonable number of them. This limitation is not dramatic as the duration of TPs depends only weakly on barrier height. If needed, our method can also be used with biased potential surfaces to accelerate the dynamics.

Experimental validation of assembly mechanism can come from direct observation, for example, by single-molecule experiments, or by perturbation, for example, by changing the thermodynamic state or the chemical composition. For the former, the simulations would make predictions of observables along the assembly routes. For the latter, the effect of the perturbation on the assembly process would have to be recapitulated in the simulations. As an example, changes in temperature altered the rate and the pathway of clathrate nucleation^21^.

Machine-guided mechanism discovery^30^ readily integrates advances in machine learning applied to force fields^19,31^, sampling^32–34^ and molecular representation^19,31,35^. Increasing computational power and advances in symbolic artificial intelligence will enable algorithms to distill ever-more-accurate mathematical descriptions of the complex processes hidden in high-dimensional data^36^. As shown here, machine-guided sampling and model validation combined with symbolic regression can support the scientific discovery process.

## Methods

### Maximum likelihood estimation of the committor function

The committor *p*_B_(**x**) is the probability that a trajectory initiated at configuration **X** with Maxwell–Boltzmann velocities reaches the (meta)stable state B before reaching A. Trajectory shooting thus constitutes a Bernoulli process. We expect to observe *n*_A_ and *n*_B_ trajectories to end in A and B, respectively, with binomial probability $p({n}_{{\mathrm{A}}},{n}_{{\mathrm{B}}}| {{{\bf{x}}}})=\binom{{{n}_{{\mathrm{A}}}+{n}_{{\mathrm{B}}}}}{{{n}_{{\mathrm{A}}}}}{(1-{p}_{{\mathrm{B}}}({{{\bf{x}}}}))}^{{n}_{{\mathrm{A}}}}{p}_{{\mathrm{B}}}{({{{\bf{x}}}})}^{{n}_{{\mathrm{B}}}}$. For *k* shooting points **x**_*i*_, the combined probability defines the likelihood ${{{\mathcal{L}}}}=\mathop{\prod }\nolimits_{i = 1}^{k}p({n}_{{\mathrm{A}}}(i),{n}_{{\mathrm{B}}}(i)| {{{{\bf{x}}}}}_{i})$. Here we ignore the correlations that arise in fast inertia-dominated transitions for trajectories shot off with opposite initial velocities^11,18^. We model the unknown committor with a parametric function and estimate its parameters **w** by maximizing the likelihood ${{{\mathcal{L}}}}$ (refs. ^5,15^). We ensure that 0 ≤ *p*_B_(**x**) ≤ 1 by writing the committor in terms of a sigmoidal activation function, ${p}_{{\mathrm{B}}}[q({{{\bf{x}}}}| {{{\bf{w}}}})]=1/(1+\exp [-q({{{\bf{x}}}}| {{{\bf{w}}}})])$. Here we model the log-probability *q*(**x**|**w**) using a neural network^15^ and represent the configuration with a vector **x** of features. For *N* > 2 states *S*, the multinomial distribution provides a model for *p*(*n*_1_, *n*_2_, . . . , *n*_*N*_|**x**), and writing the committors to states *S* in terms of the softmax activation function ensures normalization, $\mathop{\sum }\nolimits_{S = 1}^{N}{p}_{S}=1$. The loss function *l*(**w**|***θ***) used in the training is the negative logarithm of the likelihood ${{{\mathcal{L}}}}$.

### Training points from transition path sampling

TPS^4,12^ is a powerful Markov chain MC method in path space to sample TPs. The two-way shooting move is an efficient proposal move in TPS^4^. It consists of randomly selecting a shooting point **X**_SP_ on the current TP *χ* according to probability *p*_sel_(**X**_SP_|*χ*), drawing random Maxwell–Boltzmann velocities, and propagating two trial trajectories from **X**_SP_ until they reach either one of the states. Because one of the trial trajectories is propagated after first inverting all momenta at the starting point, that is, it is propagated backward in time, a continuous TP can be constructed if both trials end in different states. Given a TP *χ*, a new TP *χ*′ generated by two-way shooting is accepted into the Markov chain with probability^37^${p}_{{{{\rm{acc}}}}}({\chi }^{{\prime} }| \chi )=\min (1,{p}_{{{{\rm{sel}}}}}({{{{\bf{X}}}}}_{{{{\rm{SP}}}}}| {\chi }^{{\prime} })/{p}_{{{{\rm{sel}}}}}({{{{\bf{X}}}}}_{{{{\rm{SP}}}}}| \chi ))$. If the new path is rejected, *χ* is repeated.

Knowing the committor, it is possible to increase the rate at which TPs are generated by biasing the selection of shooting points towards the transition state ensemble^37^, that is, regions with high reactive probability *p*(TP|**X**). For the two-state case, this is equivalent to biasing towards the *p*_B_(**x**) = 1/2 isosurface defining the transition states with *q*(**x**) = 0. To construct an algorithm that selects new shooting points biased toward the current best guess for the transition state ensemble and that iteratively learns to improve its guess based on every newly observed shooting outcome, we need to balance exploration with exploitation. To this end, we select the new shooting point **X** from the current TP *χ* using a Lorentzian distribution centered around the transition state ensemble, ${p}_{{{{\rm{sel}}}}}({{{\bf{X}}}}| \chi )=1/\mathop{\sum}\limits_{{{{{\bf{x}}}}}^{{\prime} }\in \chi }[(q{({{{\bf{x}}}})}^{2}+{\gamma }^{2})/(q{({{{{\bf{x}}}}}^{{\prime} })}^{2}+{\gamma }^{2})],$ where larger values of *γ* lead to an increase of exploration. The Lorentzian distribution provides a trade-off between production efficiency and the occasional exploration away from the transition state, which is necessary to sample alternative reaction channels.

With the learned committor function, one can optimize the definition of the state boundaries. An initially tight state definition can be softened by moving the boundaries outward to, say, *p*_B_(**x**) = 0.1 and *p*_B_(**x**) = 0.9. This loosening leads to shorter TPs and speeds up the sampling.

### Real-time validation of committor model prediction

**Extended Data Fig. 9:**
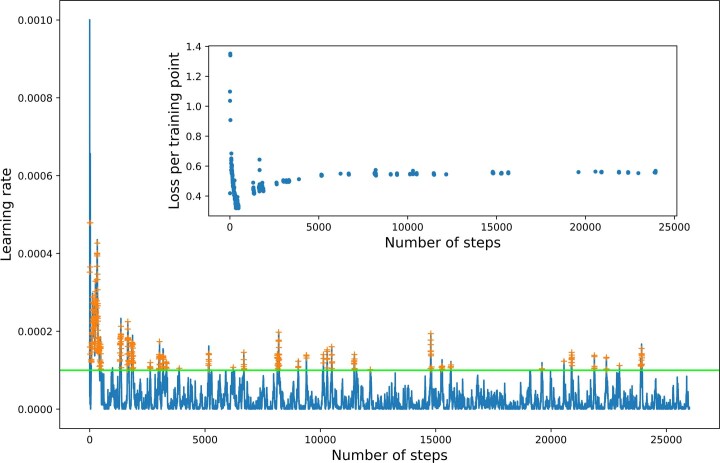
Training iterations for LiCl assembly. Training iterations for Li^+^ Cl^−^ assembly. The blue line shows the learning rate calculated from the efficiency factor at every step, orange crosses show when training actually occurred. The inset shows the training loss per shooting point for every training. Only the first 26000 Monte Carlo steps are shown. The model uses the inputs ‘SF longranged II’ and is of architecture ‘ResNet I’ (see Supplementary Tables 5 and 6). Source data

The relation between the committor and the transition probability^11^ enables us to calculate the expected number of TPs generated by shooting from a configuration **X**. We validate the learned committor on-the-fly by estimating the expected number of transitions before shooting from a configuration and comparing it with the observed shooting result. The expected number of transitions ${n}_{{{{\rm{TP}}}}}^{\exp }$ calculated over a window containing the *k* most recent two-way shooting^4^ attempts is ${n}_{{{{\rm{TP}}}}}^{\exp }=\mathop{\sum }\nolimits_{i = 1}^{k}2(1-{p}_{{\mathrm{B}}}({{{{\bf{x}}}}}_{i},i)){p}_{{\mathrm{B}}}({{{{\bf{x}}}}}_{i},i)$, where *p*_B_(**x**_*i*_, *i*) is the committor estimate for trial shooting point **X**_*i*_ at step *i* before observing the shooting result. We initiate learning when the predicted (${n}_{{{{\rm{TP}}}}}^{\exp }$) and actually generated (${n}_{{{{\rm{TP}}}}}^{{{{\rm{gen}}}}}$) number of TPs differ. We define an efficiency factor, ${\alpha }_{{{{\rm{eff}}}}}=\min (1,{(1-{n}_{{{{\rm{TP}}}}}^{{\mathrm{gen}} }/{n}_{{{{\rm{TP}}}}}^{{\mathrm{exp}} })}^{2})$, where a value of zero indicates perfect prediction (Extended Data Fig. 9). By training only when necessary, we avoid overfitting. Here we used *α*_eff_ to scale the learning rate in the gradient descent algorithm. In addition, no training takes place if *α*_eff_ is below a certain threshold (specified further below for each system).

### Neural network architectures

Molecular mechanisms can be described at different levels of resolution. One can use many high-resolution features that quantify local properties or fewer low-resolution features that measure global properties. While high-resolution features tend to be readily available, the choice of meaningful low-resolution features relies on physical understanding. With a focus on rare molecular events, we aimed to arrange features in a resolution hierarchy, going from Cartesian coordinates of atomic positions—the highest possible resolution—to a single quantity, the committor.

We designed the neural networks in this study to encourage them to learn the resolution hierarchy of features. Neural networks have shown the ability to learn low-resolution features from high-resolution ones, for example, when used in image recognition. From a practical point of view, the layer width of our networks is constantly decreasing, moving from inputs to output. In addition, as the learned features become increasingly global (and therefore less redundant) while going to deeper layers, we decrease the dropout probability moving up in the network. This is also reflected in the different architecture used for the clathrate formation where, due to the already coarse-grained and system-specific features, we used a comparatively simple pyramidal feed-forward network.

### Distilling explicit expressions for the committor

In any specific molecular process, we expect that only a few of the many degrees of freedom actually control the transition dynamics. We identify the inputs to the committor model that have the largest role in determining its output after training. To this end, we first calculate a reference loss, *l*_ref_ = *l*(**w**, **θ**), over the unperturbed training set to compare with the values obtained by perturbing every input one by one^38^. We then average the loss $l({{{\bf{w}}}},{\widetilde{{{{\bf{\uptheta }}}}}}_{i})$ over ≥100 perturbed training sets ${\widetilde{{{{\bf{\uptheta }}}}}}_{i}$ with randomly permuted values of the input coordinate *i* in the batch dimension. The average loss difference ${{\Delta }}{l}_{i}=\left\langle l({{{\bf{w}}}},{\widetilde{{{{\bf{\uptheta }}}}}}_{i})\right\rangle -{l}_{{{{\rm{ref}}}}}$ is large if the *i*th input strongly influences the output of the trained model, that is, it is relevant for predicting the committor.

In the low-dimensional subset consisting of only the most relevant inputs **z** (the ones with the highest Δ*l*_*i*_), symbolic regression generates compact mathematical expressions that approximate the full committor. Our implementation of symbolic regression is based on the Python package dcgpy^39^ and uses a genetic algorithm with a (*N* + 1) evolution strategy. In every generation, *N* new expressions are generated through random changes to the mathematical structure of the fittest expression of the parent generation. A gradient-based optimization is subsequently used to find the best parameters for every expression. The fittest expression is then chosen as parent for the next generation. The fitness of each trial expression *p*_B_(**z**) is measured by ${l}_{{{{\rm{sr}}}}}({{{{\bf{w}}}}}_{{{{\rm{sr}}}}}| {{{\bf{\uptheta }}}})\equiv -\log {{{\mathcal{L}}}}[{p}_{{\mathrm{B}}}({{{{\bf{z}}}}}_{{{{\rm{sp}}}}})]+\lambda C$, where we added the regularization term *λC* to the log-likelihood (see ‘Maximum likelihood estimation of the committor function’) to keep expressions simple and avoid overfitting. Here *λ* > 0 and *C* is a measure of the complexity of the trial expression, estimated in our case by the number of mathematical operations.

Symbolic regression will produce expressions of differing complexity depending on the regularization strength. We select expressions with a reasonable trade-off between simplicity and accuracy using a Pareto plot (Fig. 2c), in which we plot the complexity (measured as the number of mathematical operations) against the accuracy (measured as the loss on validation data). By scanning a range of *λ* values, we can identify models at the Pareto front for further analysis.

### Assembly of ion pairs in water

We investigated the formation of monovalent ion pairs in water to assess the ability of the algorithm to accurately learn the committor for transitions that are strongly influenced by solvent degrees of freedom. We used six different system set-ups (LiCl, LiI, NaCl, NaI, CsCl and CsI), each consisting of one cation and one anion in water.

All MD simulations were carried out in cubic simulation boxes using the Joung and Cheatham force field^40^ together with TIP3P^41^ water. Each simulation box contained a single ion pair solvated with 370 TIP3P water molecules. We used the openMM MD engine^42^ to propagate the equations of motion in time steps of Δ*t* = 2 fs with a velocity Verlet integrator with velocity randomization^43^ from the Python package openmmtools. After an initial NPT equilibration at constant pressure *P* = 1 bar and constant temperature *T* = 300 K, all production simulations were performed in the NVT ensemble at a constant volume *V* and a constant temperature of *T* = 300 K. The friction was set to 1 ps^−1^. Non-bonded interactions were calculated using a particle mesh Ewald scheme^44^ with a real-space cut-off of 1 nm and an error tolerance of 0.0005. Each TPS simulation (consisting of MD simulations and neural network training) was carried out on half a node using one Xeon Gold 6248 central processing unit (CPU) in conjunction with one RTX5000 GPU. In TPS, the associated and disassociated states were defined according to interionic distances (see Supplementary Table 4 for the values for each ionic species).

The committor of a configuration is invariant under global translations and rotations in the absence of external fields, and it is additionally invariant with respect to permutations of identical particles. We therefore chose to transform the system coordinates from the Cartesian space to a representation that incorporates the physical symmetries of the committor. To achieve an almost lossless transformation, we used the interionic distance to describe the solute and we adapted symmetry functions to describe the solvent configuration^45^. Symmetry functions were developed originally to describe molecular structures in neural network potentials^19,46^, but have also been successfully used to detect and describe different phases of ice in atomistic simulations^47^. These functions describe the environment surrounding a central atom by summing over all identical particles at a given radial distance. The ${G}_{i}^{2}$ type of symmetry function quantifies the density of solvent molecules around a solute atom *i* in a shell centered at *r*_s_

$${G}_{i}^{2}=\mathop{\sum}\limits_{j}{{\mathrm{e}}}^{-\eta {({r}_{ij}-{r}_{{\mathrm{s}}})}^{2}}{f}_{{\mathrm{c}}}({r}_{ij}),$$

where the sum runs over all solvent atoms *j* of a specific atom type, *r*_*ij*_ is the distance between the central atom *i* and atom *j*, and *η* controls the width of the shell. The function *f*_c_(*r*) is a Fermi cut-off defined as:

$${f}_{{\mathrm{c}}}(r)=\left\{\begin{array}{ll}{\left[1+\exp ({\alpha }_{{\mathrm{c}}}(r-{r}_{{{{\rm{cut}}}}}-1/\sqrt{{\alpha }_{{\mathrm{c}}}}))\right]}^{-1}\quad &r\le {r}_{{{{\rm{cut}}}}}\\ 0\quad &r > {r}_{{{{\rm{cut}}}}}\end{array}\right.,$$

which ensures that the contribution of distant solvent atoms vanishes. The scalar parameter *α*_c_ controls the steepness of the cut-off. The ${G}_{i}^{5}$ type of symmetry function additionally probes the angular distribution of the solvent around the central atom *i*

$${G}_{i}^{5}=\mathop{\sum}\limits_{j,k > j}{\left(1+\lambda \cos {\vartheta }_{ijk}\right)}^{\zeta }{{\mathrm{e}}}^{-\eta \left[{({r}_{ij}-{r}_{{\mathrm{s}}})}^{2}+{({r}_{ik}-{r}_{{\mathrm{s}}})}^{2}\right]}{f}_{{\mathrm{c}}}({r}_{ik}){f}_{{\mathrm{c}}}({r}_{ij}),$$

where the sum runs over all distinct solvent atom pairs, *ϑ*_*ijk*_ is the angle spanned between the two solvent atoms and the central solute atom, the parameter *ζ* is an even number that controls the sharpness of the angular distribution, and *λ* = ±1 sets the location of the minimum with respect to *ϑ*_*ijk*_ at π and 0, respectively. See Supplementary Table 5 for the parameter combinations used. We scaled all inputs to lie approximately in the range [0, 1] to increase the numerical stability of the training. In particular, we normalized the symmetry functions by dividing them by the expected average number of atoms (or atom pairs) for an isotropic distribution in the probing volume.

#### Type *G*^2^

The symmetry functions of type *G*^2^ count the number of solvent atoms in the probing volume; the normalization constant ${\langle N[{G}_{i}^{2}]\rangle }_{{{{\rm{iso}}}}}$ is therefore the expected number of atoms in the probing volume ${V}_{{{{\rm{probe}}}}}^{(2)}$

$${\langle N[{G}_{i}^{2}]\rangle }_{{{{\rm{iso}}}}}={\rho }_{{\mathrm{N}}}{V}_{{{{\rm{probe}}}}}^{\,(2)},$$

where *ρ*_N_ is the average number density of the probed solvent atom type. The exact probing volume for the *G*^2^ type can be approximated as

$$\begin{array}{lll}{V}_{{{{\rm{probe}}}}}^{(2)}&=&\int\nolimits_{0}^{\infty }{\mathrm{d}}r\int\nolimits_{0}^{\uppi }{\mathrm{d}}\theta \int\nolimits_{0}^{2\uppi }{\mathrm{d}}\phi \,{r}^{2}\sin (\theta )\exp (-\eta {(r-{r}_{{\mathrm{s}}})}^{2})\,{f}_{{\mathrm{c}}}(r)\\ &\approx &8\uppi {r}_{{\mathrm{s}}}^{2}\sqrt{2/\eta }.\end{array}$$

for small *η* and *r*_cut_ > *r*_s_.

#### Type *G*^5^

The functions of type *G*^5^ include an additional angular term and count the number of solvent atom pairs located on opposite sides of the central solute atom. The expected number of pairs ${\langle {N}_{{{{\rm{pairs}}}}}\rangle }_{{{{\rm{iso}}}}}$ can be calculated from the expected number of atoms in the probed volume ${\langle {N}_{{{{\rm{atoms}}}}}\rangle }_{{{{\rm{iso}}}}}$ as ${\langle {N}_{{{{\rm{atoms}}}}}\rangle }_{{{{\rm{iso}}}}}({\langle {N}_{{{{\rm{atoms}}}}}\rangle }_{{{{\rm{iso}}}}}-1)/2$. This expression is only exact for integer values of ${\langle {N}_{{{{\rm{atoms}}}}}\rangle }_{{{{\rm{iso}}}}}$ and can even become negative if ${\langle {N}_{{{{\rm{atoms}}}}}\rangle }_{{{{\rm{iso}}}}} < 1$. We therefore used an approximation which is guaranteed to be non-negative

$${\langle {N}_{{{{\rm{pairs}}}}}\rangle }_{{{{\rm{iso}}}}}\approx \frac{{\langle {N}_{{{{\rm{atoms}}}}}\rangle }_{{{{\rm{iso}}}}}^{2}}{2}.$$

The expected number of atoms ${\langle {N}_{{{{\rm{atoms}}}}}\rangle }_{{{{\rm{iso}}}}}$ can be calculated from the volume that is probed for a fixed solute atom and with one fixed solvent atom

$$\begin{array}{lll}{V}_{{{{\rm{probe}}}}}^{(5)}&=&{2}^{1-\zeta }\int\nolimits_{0}^{\infty }{\mathrm{d}}r\int\nolimits_{0}^{\uppi }{\mathrm{d}}\theta \int\nolimits_{0}^{2\uppi }{\mathrm{d}}\phi \,{r}^{2}\sin (\theta ){(1\pm \cos (\phi ))}^{\zeta }\exp (-\eta {(r-{r}_{{\mathrm{s}}})}^{2})\,{f}_{{\mathrm{c}}}(r)\\ &=&{2}^{1-\zeta }{V}_{{{{\rm{probe}}}}}^{(2)}\frac{(2\zeta -1)!!}{\zeta !}\end{array}$$

With the expectation that most degrees of freedom of the system do not control the transition, we designed neural networks that progressively filter out irrelevant inputs and build a highly nonlinear function of the remaining ones. We tested three different pyramidal neural network architectures ‘ResNet I’, ‘ResNet II’ and ‘SNN’, where names containing ‘ResNet’ indicate the use of residual units^48,49^ and ‘SNN’ a self normalizing neural network architecture^50^ (see Supplementary Tables 6–8 for the exact architectures used). The best performing architecture is ‘ResNet I’ (see Supplementary Data File 1 for performance comparison of the different architectures for all ionic systems). ResNet I used a pyramidal stack of five pre-activation residual units, each with four hidden layers. The number of hidden units per layer is reduced by a constant factor $f={(10/{n}_{{\mathrm{in}}})}^{1/4}$ after every residual unit block and decreases from *n*_in_ = 221 in the first unit to 10 in the last. In addition, a dropout of 0.1*f*^*i*^, where *i* is the residual unit index ranging from 0 to 4, is applied after every residual block. Optimization of the network weights is performed using the Adam gradient descent^51^. For all architectures, training was performed after every third TPS MC step for one epoch with a learning rate of lr = *α*_eff_10^−3^, if lr ≥ 10^−4^. The expected efficiency factor *α*_eff_ was calculated over a window of *k* = 100 TPS steps. We performed all deep learning with custom written code based on Keras^52^. The TPS simulations were carried out using a customized version of openpathsampling^53,54^ together with our own Python module.

For the transfer training, the last layer with a single neuron (that is, the log predictor) of a model originally trained on LiCl was randomized and all other weights were kept fixed during the subsequent training on the shooting data for the other ionic species (LiI, NaCl, NaI, CsCl and CsI). Training was performed using the Adam optimizer with a learning rate of lr = 2.5 × 10^−5^. The test loss was calculated after every epoch on 20% of the data used as test set. The training was stopped when no decrease in the test loss was observed for more than 1,000 epochs. The model with the best test loss was then used.

For the extrapolation in chemical space (Extended Data Fig. 3), we set up a multi-ion neural network of architecture ‘ResNet I’. The model was trained on the shooting results for different pairs of ionic species simultaneously, as specified. It used the coordinates from the set ‘SF shortranged’ together with the *ϵ* and *σ* Lennard-Jones parameters of the force field to distinguish the different ionic species. Training was performed with the Adam optimizer (lr = 10^−3^) using 10% of the data as test set. The training was terminated if the test loss did not decrease for 1,000 epochs and the model with the best test loss was then used.

We selected the seven most relevant coordinates identified by the multi-ion neural network as inputs for the multi-ion symbolic regressions (Supplementary Tables 9–12). We used between 3 and 7 of these most relevant coordinates for independent symbolic regression runs using the regularization values *λ* = 0.001, *λ* = 0.0001 and *λ* = 0.00001. We then selected the expression reported in Fig. 2d using the Pareto plot in Fig. 2c.

We also selected the five most relevant coordinates identified from a neural network trained on LiCl for symbolic regression runs (Extended Data Fig. 4b–d). We regularized the produced expressions by penalizing the total number of elementary mathematical operations with *λ* = 10^−6^ and *λ* = 10^−7^.

The contributions of each atom to the committor in a particular system **X** (Fig. 1b) was calculated as the magnitude of the gradient of the reaction coordinate *q*(**x**) with respect to its Cartesian coordinates. All gradient magnitudes were scaled with the inverse atom mass.

### Nucleation of methane clathrates

The learning algorithm was applied to an existing TPS dataset of methane-clathrate nucleation initially produced for ref. ^21^. It contains data for simulations carried out at four different temperatures *T* = 270 K, 275 K, 280 K and 285 K (see Supplementary Table 14 for details). New simulations were performed to obtain the sampled committor values used in the validation. All committor simulations were performed with OpenMM 7.1.1^42^ on NVIDIA GeForce GTX TITAN 1080Ti GPUs, shooting between 6 and 18 trajectories per configuration using the same simulation protocol as in ref. ^21^.

We used 22 different features to describe size, crystallinity, structure and composition of the growing methane-hydrate crystal nucleus (Supplementary Table 1). In addition to the features describing molecular configurations, we used temperature as an input to the neural networks and the symbolic regression. In a pyramidal feed-forward network with 9 layers, we reduced the number of units per layer from 23 at the input to one in the last layer (Supplementary Table 15). The network was trained with the Adam optimizer with learning rate lr = 10^−3^ on the existing TPS data for all temperatures, leaving out 10% of the shooting points as test data. We stopped the training after the loss on the test set did not decrease for 10,000 epochs and used the model with the best test loss. All neural network training was performed on a RTX6000 GPU. We used the three most relevant coordinates as inputs for symbolic regression runs with a penalty on the total number of elementary mathematical operations using *λ* = 10^−5^.

### Polymer folding

We applied our machine learning algorithm on existing shooting data of polymer crystallization^24,25^. We used two different sets of features to describe the transition, a set of 35 low-resolution (coarse-grained) features that has also been used in previous work and a set of high-resolution features describing each polymer bead on its own. The low-resolution features contain a number of global measures such as the potential energy *U* and the Steinhardt bond-order parameters *Q*_4_ and *Q*_6_, descriptions of the local environment of selected polymer particles, various measures describing the structure of the polymer by counting chains and loops, and some selected distances (see Supplementary Table 16 for an exhaustive list). The high-resolution feature set consists of the number of connections, neighbors and the neighbor-averaged Lechner–Dellago Steinhardt bond-order parameters^55^ for each polymer bead, that is, each configuration corresponds to a feature vector with 3 × 128 = 384 entries.

For both the high-resolution and the low-resolution description, we used pyramid shaped neural networks (Supplementary Tables 17 and 18). In both cases, training was performed using the Adam gradient descent method with a learning rate lr = 10^−3^ using 20% of the data as test data. The models were saved and the test loss was calculated after every epoch. The training was stopped if the test loss did not decrease for 10,000 epochs. The model with the lowest test loss was then used as the final trained model. All neural network training was performed on an RTX6000 GPU.

We used between two and five of the five most relevant low-resolution features as inputs in symbolic regression runs (Supplementary Tables 19–22). We regularized by penalizing the number of elementary mathematical operations with *λ* = 10^−2^, 10^−3^, 10^−4^, 10^−5^ and 10^−6^.

### Mga2 transmembrane dimer assembly in lipid membrane

We used the coarse-grained Martini force field (v2.2)^56–59^ to describe the assembly of the alpha-helical transmembrane homodimer Mga2. All MD simulations were carried out with GROMACS v4.6.7^60–63^ with an integration timestep of Δ*t* = 0.02 ps, using a cubic simulation box containing the two identical 30-amino-acid-long alpha helices in a lipid membrane made of 313 1-palmitoyl-2-oleoyl-glycero-3-phosphocholine (POPC) molecules. The membrane spans the box in the *x*–*y* plane and was solvated with water (5,348 water beads) and NaCl ions corresponding to a concentration of 150 mM (58 Na^+^, 60 Cl^−^). A reference temperature of *T* = 300 K was enforced using the v-rescale thermostat^64^ with a coupling constant of 1 ps separately on the protein, the membrane and the solvent. A pressure of 1 bar was enforced separately in the *x*–*y* plane and in *z* using a semiisotropic Parrinello–Rahman barostat^65^ with a time constant of 12 ps and compressibility of 3 × 10^−4^ bar^−1^. Each MD simulation was carried out on a single compute node with two E5-2680-v3 CPUs and 64 GB memory. All neural network training was performed on an RTX6000 GPU.

To describe the assembly of the Mga2 homodimer, we used 28 interhelical pairwise distances between the backbone beads of the two helices together with the total number of interhelical contacts, the distance between the helix centers of mass and a number of hand-tailored features describing the organization of lipids around the two helices (Supplementary Table 2). To ensure that all network inputs lie approximately in [0, 1], we used the sigmoidal function $f(r)={(1-(r/{R}_{0})^{6})}/{(1-(r/{R}_{0})^{12})}$ with *R*_0_ = 2 nm for all pairwise distances, while we scaled all lipid features using the minimal and maximal values taken along the transition. The assembled and disassembled states are defined as configurations with ≥130 interhelical contacts and with helix–helix center-of-mass distances *d*_CoM_ ≥ 3 nm, respectively.

The neural network used to fit the committor was implemented using Keras^52^ and consisted of an initial 3-layer pyramidal part in which the number of units decreases from the 36 inputs to 6 in the last layer using a constant factor of (6/36)^1/2^ followed by 6 residual units^48,49^, each with 4 layers and 6 neurons per layer (Supplementary Table 23). A dropout of 0.01 is applied to the inputs and the network is trained using the Adam gradient descent protocol with a learning rate of lr = 0.0001 (ref. ^51^).

To investigate the assembly mechanism of Mga2, we performed machine-guided sampling in parallel on multiple nodes of a high-performance computer cluster. We ran 500 independent TPS chains guided by the current committor model. The 500 TPS simulations were initialized with random initial TPs. The neural network used to select the initial shooting points was trained on preliminary shooting attempts (8,044 independent shots from 1,160 different points). After two rounds (two steps in each of the 500 independent TPS chains), we updated the committor model by training on all new data. We retrained again after the sixth round. No further training was required, as indicated by consistent numbers of expected and observed counts of TPs. We performed another 14 rounds for all 500 TPS chains to collect TPs (Fig. 5b). Shooting point selection, TPS set-up and neural network training were fully automated in Python code using MDAnalysis^66,67^, numpy^68^ and our custom Python package.

The input importance analysis revealed the total number of contacts *n*_contacts_ as the single most important input (Extended Data Fig. 7). However, no expression generated by symbolic regression as a function of *n*_contacts_ alone was accurate in reproducing the committor. It is likely that *n*_contacts_ is used by the trained network only as a binary switch to distinguish the two different regimes—close to the bound or to the unbound states. By restricting the input importance analysis to training points close to the unbound state, we found that the network uses various interhelical contacts that approximately retrace a helical pattern (Extended Data Fig. 7). We performed symbolic regression on all possible combinations made by one, two or three of the seven most important input coordinates (Supplementary Table 3). The best expressions in terms of the loss were selected using validation committor data that had not been used during the optimization. This validation set consisted of committor data for 516 configurations with 30 trial shots each and 32 configurations with 10 trial shots.

To asses the variability in the observed reaction mechanisms, we performed a hierarchical clustering of all TPs projected into the plane defined by the contacts 9 and 22, which enter the most accurate parametrization generated by symbolic regression. We then used dynamic time warping^69^ to calculate the pairwise similarity between all TPs for the clustering, which we performed using the scipy clustering module^70,71^. The path density plots (Fig. 5f,g) were histogrammed according to the number of paths, not the number of configurations, that is, the counter of each cell visited by a particular path was incremented by one for this path.

### Supplementary information

Supplementary Information Supplementary Tables 1–23.

Supplementary Data 1 Efficiency of ion TPS simulations: number of accepted, generated and expected transitions per Monte Carlo step for all machine learning-assisted TPS simulations on all ionic species. The difference shown in the last column is between expected and generated transitions, that is, (expected − generated)/steps. The values for the initial (final) phase were calculated over the first (last) 1,000 MC steps, the value for the full simulation is over all 100,000 MC steps.

### Source data

Source Data Fig. 1 Statistical source data for Fig. 1b–e.

Source Data Fig. 2 Statistical source data for Fig. 2a,c,e.

Source Data Fig. 3 Statistical source data for Fig. 3b,c.

Source Data Fig. 4 Statistical source data for Fig. 4a,b.

Source Data Fig. 5 Statistical source data for Fig. 5b,c,e–g.

Source Data Extended Data Fig. 1 Statistical source data for Extended Data Fig. 1.

Source Data Extended Data Fig. 2 Statistical source data for Extended Data Fig. 2.

Source Data Extended Data Fig. 3 Statistical source data for Extended Data Fig. 3.

Source Data Extended Data Fig. 4 Statistical source data for Extended Data Fig. 4a,b,d.

Source Data Extended Data Fig. 5 Statistical source data for Extended Data Fig. 5.

Source Data Extended Data Fig. 6 Statistical source data for Extended Data Fig. 6.

Source Data Extended Data Fig. 7 Statistical source data for Extended Data Fig. 7.

Source Data Extended Data Fig. 8 Statistical source data for Extended Data Fig. 8.

Source Data Extended Data Fig. 9 Statistical source data for Extended Data Fig. 9.

## Data Availability

Training set data and files to setup molecular dynamics simulations for the assembly of LiCl are included in the Code Ocean capsule^72^. Data to reproduce this study for all remaining systems (all remaining ions, polymer, clathrate, and MGA2 transmembrane dimer) are publicly available in a Zenodo repository^73^. Source data are provided with this paper.

## Code availability

An executable version of the ‘Artificial Intelligence for Molecular Mechanism Discovery’ (AIMMD) code is available in the Code Ocean software capsule^72^. The AIMMD code is also available at https://github.com/bio-phys/aimmd as git repository.
