# Supplementary material for: Machine-guided path sampling to discover mechanisms of molecular self-organization
Source: Nat Comput Sci. 2023 Apr 24;3(4):334–45. doi: 10.1038/s43588-023-00428-z (PMC10766509; doi:10.1038/s43588-023-00428-z)
Supplement: Supplementary file 1 — Supplementary Tables 1–23. [file 43588_2023_428_MOESM1_ESM.pdf]

nature computational science

Article https://doi.org/10.1038/s43588-023-00428-z

Machine-guided path sampling to discover mechanisms of molecular self-organization

In the format provided by the authors and unedited

Supplementary Table 1. Overview of the features used to describe the methane clathrate nucleation. Features are grouped by category. The indices indicate the position in the feature vectors used as input in the neural networks and symbolic regression. Mutually coordinated guest (MCG) numbers were calculated as in Ref. 1.

Category	Name	Index	Definition
Methanes in nucleus	MCG	0	Total number of methanes in the largest cluster[2]
	N_{sm_1}	11	Methanes (in MCG) with only 1 methane neighbor within 0.9 nm
	N_{sm_2}	12	Methanes (in MCG) with 1 or 2 methane neighbor within 0.9 nm
	N_{cm_1}	13	Core methanes: MCG minus N_{sm_1}
	N_{cm_2}	14	Core methanes: MCG minus N_{sm_2}
Waters molecules in the nucleus	N_{w_2}	3	Number of waters with 2 MCG carbons within 0.6 nm
	N_{w_3}	2	Number of waters with 3 MCG carbons within 0.6 nm
	N_{w_4}	1	Number of waters with 4 MCG carbons within 0.6 nm
	N_{sw_2-3}	5	Surface water molecules 1: $N_{w\_2} -$ N_{w_3}
	N_{sw_3-4}	4	Surface water molecules 2: $N_{w\_3} -$ N_{w_4}
Structure of the nucleus	$5^{12}6^2$ cages	8	Cages with 12 planar five-rings and 2 planar six-rings
	5^{12} cages	9	Cages with 12 planar five-rings
	$5^{12}6^3$ cages	17	Cages with 12 planar five-rings and 3 planar six-rings
	$5^{12}6^4$ cages	18	Cages with 12 planar five-rings and 3 planar six-rings
	$4^15^{12}6^2$ cages	19	Cages with 1 planar four-ring, 12 five-rings and 2 six-rings
	$4^15^{12}6^3$ cages	20	Cages with 1 planar four-ring, 12 five-rings and 3 six-rings
	$4^15^{12}6^4$ cages	21	Cages with 1 planar four-ring, 12 five-rings and 4 six-rings
	Cage Ratio	10	$5^{12}6^2$ cages divided by 5^{12} cages
	R_g	7	Radius of gyration of the nucleus
Global Crystallinity	F4	6	Average of 3 times the cosine of the dihedral angle between two neighboring waters.[3]

Supplementary Table 2. Features used to describe Mga2 transmembrane assembly. The features are grouped by category. The subscripts in the feature names in column 2 indicate the position in the feature vectors used as input for neural networks and symbolic regression. The third column gives a description of the features.

Category	Name	Definition
Pairwise interhelical contacts	x_0 - x_{27}	$x_i(r_i) = \frac{1-(r_i/(2\text{ nm}))^6}{1-(r_i/(2\text{ nm}))^{12}}$, where r_i is the distance between the ith residue on each helix; index 0 corresponds to ASN1034, index 28 to GLN1061
Global conformation	$x_{28} = n_{\text{contacts}}$	$n_{\text{contacts}}(\mathbf{r}) = \sum_{i=0}^{27} \frac{1-(r_i-0.07\text{ nm})/(0.7\text{ nm}))^6}{1-(r_i-0.07\text{ nm})/(0.7\text{ nm}))^{30}}$
	$x_{29} = \alpha_{tilt}$	Angle between the first principal moments of inertia of the two helices
	$x_{30} = d_{CoM}$	Center of mass distance between the two helices in the plane of the membrane
Lipid collective variables	$x_{31} = \text{CV}_{lip1}$	Number of lipid tails crossing the helix-helix interface
	$x_{32} = \text{CV}_{lip2}$	Number of lipid molecules that cross the interface with both tails
	$x_{33} = \text{CV}_{lip3}$	Number of lipid molecules with center of mass in the helix-helix interface
	$x_{34} = \text{CV}_{lip4}$	Number of lipid molecules with the headgroup in the helix-helix interface
	$x_{35} = \text{CV}_{lip5}$	Number of lipid molecules with their headgroup in the interface and the tails spread in opposite directions

Supplementary Table 3. Mga2 symbolic regression results for each possible combination of two coordinates out of the seven most relevant inputs. Combinations including n_{contacts} are omitted due to their low predictive power.

Validation loss	Expression
0.53004	$q_B(x_9, x_{22}) = -\exp(x_9^2)\log(x_9 - \frac{x_9}{\log(x_{22})})$
0.54033	$q_B(x_1, x_{22}) = (\exp(x_{22}) + 0.637)(-0.0557\exp(2x_{22}) - \log(x_{22} + x_1))$
0.53687	$q_B(x_9, x_{26}) = -x_{26} - \frac{x_{26}}{0.26/x_9 - \log(x_9)} + \log(0.26/x_9) + 1.29$
0.54909	$q_B(x_9, x_{23}) = \left(\frac{9.07x_9}{9.07x_9 - 25.9} - 1.4\right)(x_{23} + 2x_9 - 1.4)$
0.55821	$q_B(x_1, x_{20}) = 2.54 - 2.77x_{20} - 2x_1 - 0.287\exp(x_1)$
0.55709	$q_B(x_1, x_{26}) = (\exp(-2.07x_1) - x_{26})\log(21.3\exp(-x_1(1.07 - x_1) - 0.00272x_1))$
0.56051	$q_B(x_9, x_{20}) = 2.67 - \exp(x_{20}) - \frac{7.29}{-1.49 + 4.13/x_9}$
0.55964	$q_B(x_1, x_{23}) = 0.00426(-10x_{23} - 64.4\exp(x_{23}) + 924)\exp(-x_1) - 1.43\exp(x_{23})$
0.57002	$q_B(x_1, x_9) = -\log\left(-0.0626 - \frac{0.816}{\log(x_9)}\right)$
0.5956	$q_B(x_{22}, x_{26}) = 1.24 - \frac{2.76x_{22}^2}{x_{26}}$
0.58008	$q_B(x_{20}, x_{22}) = -0.496\exp(x_{20} + x_{22})\log(x_{20} + x_{22})$
0.59576	$q_B(x_{22}, x_{23}) = -(0.605\exp(x_{22}) - 0.0956)\log(x_{22}\exp(x_{22})) - 0.282$
0.59914	$q_B(x_{20}, x_{26}) = \frac{\log(2x_{20})}{x_{20} + x_{26} - 2.3}$
0.5975	$q_B(x_{20}, x_{23}) = 1.73 - \exp(x_{20}) + \frac{0.00635x_{20}(x_{20} + 6.11)}{\log(x_{23})}$
0.61409	$q_B(x_{23}, x_{26}) = \exp(-5.89x_{23} + \frac{0.0193}{x_{23}}) - \frac{8.31x_{23}\exp(\exp(x_{26}))}{-2.72 + 78.7/x_{23}^2}$

Supplementary Table 4. State definitions used in ion assembly transition path sampling simulations. Column 1 lists the different ion pairs, and columns 2 and 3 the ranges of the interionic distance r_{ion} defining the assembled and disassembled states, respectively.

Ion pair	assembled state	disassembled state
Li^+Cl^-	$r_{\text{ion}} \leq 0.23$ nm	$r_{\text{ion}} \geq 0.48$ nm
Li^+I^-	$r_{\text{ion}} \leq 0.26$ nm	$r_{\text{ion}} \geq 0.53$ nm
Na^+Cl^-	$r_{\text{ion}} \leq 0.27$ nm	$r_{\text{ion}} \geq 0.53$ nm
Na^+I^-	$r_{\text{ion}} \leq 0.31$ nm	$r_{\text{ion}} \geq 0.59$ nm
Cs^+Cl^-	$r_{\text{ion}} \leq 0.34$ nm	$r_{\text{ion}} \geq 0.60$ nm
Cs^+I^-	$r_{\text{ion}} \leq 0.38$ nm	$r_{\text{ion}} \geq 0.68$ nm

Supplementary Table 5. Sets of symmetry function parameters used to describe the ionic systems including solvent degrees of freedom. The same cutoff value, r_{cut}, is used for all symmetry functions (SFs) in each set. Note that each value of r_s is combined with all values for η, ζ and λ from subsequent rows, for SFs of type G^5 this results in a total of 10 different parameter combinations for each value of r_s.

Symmetry function set			Symmetry function type			
			$G^2(r_s, \eta)$	$G^5(r_s, \eta, \zeta, \lambda)$		
	r_{cut} [nm]	r_s [nm]	η	η	ζ	λ
**SF longranged I** (66 functions per central atom per solvent atom type)	1	0.1<br>0.25<br>0.4<br>0.55<br>0.7<br>0.85	200	120	1, 2, 4, 16, 64	+1, -1
**SF longranged II** (55 functions per central atom per solvent atom type)	1	0.25<br>0.4<br>0.55<br>0.7<br>0.85	200	120	1, 2, 4, 16, 64	+1, -1
**SF shortranged** (44 functions per central atom per solvent atom type)	0.8	0.1<br>0.25<br>0.4<br>0.55	200	120	1, 2, 4, 16, 64	+1, -1

Supplementary Table 6. “ResNet I” architectures used for ion pair assembly. The rows show the number of units for Linear and Resunit layers, the droput fraction for dropout layers. In each residual unit we used the element-wise ELU as activation function. The linear layer only reduces the width and uses no activation function.

Layer name	Input descriptor set		
	SF longranged I	SF longranged II	SF shortranged
Input dimension	265	221	177
Linear1	265	221	177
Dropout1	0.1	0.1	0.1
Resunit1	$[265] \times 4$	$[221] \times 4$	$[177] \times 4$
Linear2	116	101	86
Dropout2	0.05	0.05	0.05
Resunit2	$[116] \times 4$	$[101] \times 4$	$[86] \times 4$
Linear3	51	47	42
Dropout3	0.02	0.02	0.02
Resunit3	$[51] \times 4$	$[47] \times 4$	$[42] \times 4$
Linear4	22	21	20
Dropout4	0.01	0.01	0.01
Resunit4	$[22] \times 4$	$[21] \times 4$	$[20] \times 4$
Linear5	9	10	9
Dropout5	0.004	0.004	0.004
Resunit5	$[9] \times 4$	$[10] \times 4$	$[10] \times 4$
Log predictor	1	1	1

Supplementary Table 7. Self-normalizing neural network ("SNN") architectures used for ion pair assembly. The rows show the number of units per layer for Linear, for Alpha dropout the dropout fraction.

	Input descriptor set	
Layer name	SF longranged I	SF shortranged
Input dimension	265	177
Linear1 + SELU1	265	177
Alpha dropout1	0.2	0.2
Linear2 + SELU2	137	99
Alpha dropout2	0.104	0.104
Linear3 + SELU3	71	56
Alpha dropout3	0.054	0.054
Linear4 + SELU4	37	31
Alpha dropout4	0.028	0.028
Linear5 + SELU5	19	17
Alpha dropout5	0.0145	0.0145
Linear6 + SELU6	9	10
Alpha dropout6	0.008	0.008
Log predictor	1	1

Supplementary Table 8. "ResNet II" architectures used for ion pair assembly. The rows show the number of units for Resunit layers, the droput fraction for dropout layers. The residual units used the element-wise ELU activation function.

	Input descriptor set	
Layer name	SF longranged I	SF shortranged
Input dimension	265	177
Linear1 + ELU1	265	177
Dropout1	0.2	0.2
Linear2 + ELU2	116	86
Dropout2	0.09	0.09
Linear3 + ELU3	51	42
Dropout3	0.04	0.04
Linear4 + ELU4	22	20
Dropout4	0.02	0.02
Linear5 + ELU5	9	9
Dropout5	0.01	0.01
Resunit1	$[10] \times 4$	$[10] \times 4$
Resunit2	$[10] \times 4$	$[10] \times 4$
Resunit3	$[10] \times 4$	$[10] \times 4$
Log predictor	1	1

Supplementary Table 9. Seven most relevant input descriptors from a neural network simultaneously trained on all ionic species. As input coordinates the network is using the input set "SF shortranged" plus Lennard-Jones parameters ϵ and σ to distinguish between the ionic species. The network is of architecture "ResNet I". The descriptors were ordered from most relevant to least relevant. This input ordering is used for the symbolic regressions in Supplementary Tables 10, 11 and 12.

Formula sign	Collective variable definition	Unit	Minimum	Maximum	Max - Min
r_{ion}	Interionic distance	nm	0.208620	0.693789	0.485168
σ	$\sigma = (\sigma_{\text{cation}} + \sigma_{\text{anion}})/2$, effective Lennard-Jones parameter	nm	0.315200	0.430838	0.115639
x_7	$G^5_{\text{Cation}}(\eta = 120, r_s = 0.1 \text{ nm}, \zeta = 2, \lambda = -1)$[O of HOH]		$9.39807 \cdot 10^{-6}$	2.66798	2.66797
x_0	$G^2_{\text{Cation}}(\eta = 200, r_s = 0.1 \text{ nm})$[O of HOH]		$3.15353 \cdot 10^{-5}$	1.05399	1.05396
x_{15}	$G^5_{\text{Cation}}(\eta = 120, r_s = 0.25 \text{ nm}, \zeta = 1, \lambda = -1)$[O of HOH]		0.0538372	1.21434	1.16050
x_1	$G^2_{\text{Cation}}(\eta = 200, r_s = 0.25 \text{ nm})$[O of HOH]		0.164460	1.33434	1.16988
x_9	$G^5_{\text{Cation}}(\eta = 120, r_s = 0.1 \text{ nm}, \zeta = 4, \lambda = -1)$[O of HOH]		8.45728	2.82153	2.82152

Supplementary Table 10. Selected multi-ion symbolic regression results using the first $n_{in} = 3$ and $n_{in} = 4$ most relevant descriptors (see Supplementary Table 9) as inputs. Only expressions with a validation loss ≤ 0.605 and operation count ≤ 15 are reported. Expressions were sorted from lowest to highest validation loss (top to bottom) for each regularization value λ separately. The frequency is given per optimization (combination of n_{in} and λ), e.g., 1/6 means that a total of six independent optimization runs were performed for this combination and that the given expression was found in one of them. Note that many expressions were also found for other combinations of n_{in} and λ. All factors were rounded to 4 significant digits.

n_{in}	λ	$\mathcal{L}_{\text{val}}$	$\mathcal{C}$	Frequency	Final expression
3	0.001	0.604177	9	1/6	$q_B = r_{\text{ion}}(25.47 - 17.51\sigma) + \sigma(18.84x_7 - 20.92) - 5.303x_7$
		0.604494	8	2/6	$q_B = 19.37r_{\text{ion}} + \sigma(24.20x_7 - 29.64) - 7.153x_7 + 3.062$
	0.0001	0.603958	11	1/5	$q_B = r_{\text{ion}}(28.17 - 24.17\sigma) - 20.80\sigma + 1.158x_7 - 618877135401\exp(-86.20\sigma)$
		0.604177	9	3/5	$q_B = r_{\text{ion}}(25.47 - 17.51\sigma) + \sigma(18.84x_7 - 20.92) - 5.303x_7$
	0.00001	0.603652	13	1/5	$q_B = r_{\text{ion}}(46.93 - 69.77\sigma) - 8.545\exp\left(\frac{0.08175x_7}{r_{\text{ion}}}\right) + \frac{x_7(4.988\sigma - 0.4464)}{r_{\text{ion}}}$
		0.603961	11	1/5	$q_B = r_{\text{ion}}(35.37 - 42.83\sigma) + \sigma(11.03x_7 - 7.878) - 5.112\exp(0.3727x_7)$
		0.603997	13	1/5	$q_B = \frac{7.285r_{\text{ion}}}{\sigma} - 8.161\sigma + 9.274x_7 - 4.996\exp(0.1770x_7) - \frac{2.415x_7}{\sigma}$
		0.604355	13	1/5	$q_B = 19.078r_{\text{ion}} - 34.45\sigma + 1.791\exp(1.069\exp(0.1726x_7 - 1842224\exp(-50.54\sigma)))$
4	0.001				
	0.0001	0.604177	9	2/5	$q_B = r_{\text{ion}}(25.47 - 17.51\sigma) + \sigma(18.84x_7 - 20.92) - 5.303x_7$
		0.604696	12	1/5	$q_B = 75.46r_{\text{ion}}\exp(-3.645\sigma) + 6.451x_7 - 18.14x_7\exp(-3.645\sigma) - 7.929$
	0.00001	0.604038	14	1/5	$q_B = \frac{8.678r_{\text{ion}}}{\sigma} + 20.94x_0 - 11.62 + \frac{-7.570x_0 + 0.5676x_7 - 0.6066\log(r_{\text{ion}}) + 0.2232}{\sigma}$
		0.604177	9	1/5	$q_B = r_{\text{ion}}(25.47 - 17.51\sigma) + \sigma(18.84x_7 - 20.92) - 5.303x_7$

Supplementary Table 11. Selected multi-ion symbolic regression results using the first $n_{in} = 5$ and $n_{in} = 6$ most relevant descriptors (see Supplementary Table 9) as inputs. Only expressions with a validation loss ≤ 0.605 and operation count ≤ 15 are reported. Expressions were sorted from lowest to highest validation loss (top to bottom) for each regularization value λ separately. The frequency is given per optimization (combination of n_{in} and λ), e.g., 1/6 means that a total of six independent optimization runs were performed for this combination and that the given expression was found in one of them. Note that many expressions were also found for other combinations of n_{in} and λ. All factors were rounded to 4 significant digits.

n_{in}	λ	$\mathcal{L}_{\text{val}}$	$\mathcal{C}$	Frequency	Final expression
5	0.001	0.604590	9	1/7	$q_B = 18.10r_{\text{ion}} + \sigma(20.27x_7 - 21.95) - 5.403x_7 + 1.187x_{15}$
	0.0001	0.603243	13	1/6	$q_B = 18.22r_{\text{ion}} - 22.17\sigma + 1.922x_7 + 1.302x_{15} - 245497\exp(2.728x_0 - 44.05\sigma)$
		0.603639	12	1/6	$q_B = 14.44x_0 + 19.12r_{\text{ion}} - 22.36\sigma + \frac{-3.861x_0 + 0.008308\exp(4.585x_{15})}{\sigma}$
		0.603875	11	1/6	$q_B = r_{\text{ion}}(21.56 - 8.669\sigma) + \sigma(17.80x_7 - 21.33) - 4.771x_7 + 0.6775x_{15}$
		0.604973	9	1/6	$q_B = 18.92r_{\text{ion}} + \sigma(34.68x_0 - 22.94) - 8.721x_0 + 1.192x_{15}$
	0.00001	0.604608	11	1/5	$q_B = 19.03r_{\text{ion}} + 23.91\sigma - 2.224x_7 - 5.772\exp(2.868\sigma - 0.2668x_7)$
6	0.001	0.604590	9	1/7	$q_B = 18.10r_{\text{ion}} + \sigma(20.27x_7 - 21.95) - 5.403x_7 + 1.187x_{15}$
	0.0001	0.602661	12	1/5	$q_B = 18.43r_{\text{ion}} - \sigma(272.91x_7 + 22.54) + 189.84x_7 - \frac{32.43x_7}{\sigma} + 1.490x_{15}$
		0.603149	12	1/5	$q_B = 17.90r_{\text{ion}} - 20.79\sigma + 7.017x_7 + 0.0088\exp(5.687x_{15}) - \frac{1.889x_7}{\sigma}$
		0.603408	12	1/5	$q_B = 18.32r_{\text{ion}} - 22.31\sigma + 1.319x_{15} + 1.710x_7\exp(-18229814739461300\exp(-120.53\sigma))$
	0.00001	0.604098	13	1/4	$q_B = r_{\text{ion}}(25.00 - 24.03\sigma) + \sigma(8.581x_7 - 14.12) - 2.962\exp(-1.746r_{\text{ion}} + 0.6233x_7)$

Supplementary Table 12. Selected multi-ion symbolic regression results using the first $n_{in} = 7$ most relevant descriptors (see Supplementary Table 9) as inputs. Only expressions with a validation loss ≤ 0.605 and operation count ≤ 15 are reported. Expressions were sorted from lowest to highest validation loss (top to bottom) for each regularization value λ separately. The frequency is given per optimization (combination of n_{in} and λ), e.g., 1/6 means that a total of six independent optimization runs were performed for this combination and that the given expression was found in one of them. Note that many expressions were also found for other combinations of n_{in} and λ. All factors were rounded to 4 significant digits.

n_{in}	λ	$\mathcal{L}_{\text{val}}$	$\mathcal{C}$	Frequency	Final expression
7	0.001	0.603514	9	1/6	$q_B = 18.00 r_{\text{ion}} + \sigma\left(16.59x_7 - 20.66\right) - x_0\left(3.338x_9 + 4.463\right)$
	0.0001	0.602186	14	1/4	$q_B = 18.04 r_{\text{ion}} - 21.18\sigma - 1.590x_1 + 10.90x_7 + \dfrac{1.123x_{15} - 2.633x_7 - 0.5173x_9}{\sigma}$
		0.604796	11	1/4	$q_B = 18.63 r_{\text{ion}} - 0.4347\exp\left(5.773\sigma - 0.6643x_7\right) - 4.471\exp\left(-0.2093x_{15}\right)$
	0.00001	0.603920	11	1/4	$q_B = 18.42 r_{\text{ion}} + 0.9773x_{15} - 1.256x_9 - 3.367\exp\left(2.417\sigma - 0.4924x_7\right)$

Supplementary Table 13. Input attribution analysis for lithium-chloride ion-pair formation. Definition of the ten most relevant input coordinates as found for the "SF longranged II" set of symmetry functions, listed in decreasing order of importance.

Formula sign	Collective variable definition	Unit	Minimum	Maximum	Max - Min
r_{LiCl}	Distance between Li^+ and Cl^-	nm	0.213732	0.513406	0.299674
x_{12}	$G^5_{\text{Li}}(\eta = 120.0, r_s = 0.25, \zeta = 16, \lambda = -1.0)$[O of HOH]		0.0207623	2.48317	2.46240
x_8	$G^5_{\text{Li}}(\eta = 120.0, r_s = 0.25, \zeta = 2, \lambda = -1.0)$[O of HOH]		0.143737	0.814009	0.670271
x_{14}	$G^5_{\text{Li}}(\eta = 120.0, r_s = 0.25, \zeta = 64, \lambda = -1.0)$[O of HOH]		0.00147766	6.55636	6.55488
x_{55}	$G^2_{\text{Cl}}(\eta = 200.0, r_s = 0.25)$[O of HOH]		0.235621	0.842196	0.606575
x_6	$G^5_{\text{Li}}(\eta = 120.0, r_s = 0.25, \zeta = 1, \lambda = -1.0)$[O of HOH]		0.127523	0.685849	0.558326
x_9	$G^5_{\text{Li}}(\eta = 120.0, r_s = 0.25, \zeta = 4, \lambda = 1.0)$[O of HOH]		0.0312017	0.420071	0.388869
x_{173}	$G^5_{\text{Cl}}(\eta = 120.0, r_s = 0.25, \zeta = 2, \lambda = -1.0)$[H of HOH]		0.226796	0.944836	0.718040
x_{178}	$G^5_{\text{Cl}}(\eta = 120.0, r_s = 0.25, \zeta = 64, \lambda = 1.0)$[H of HOH]		0.113978	0.821783	0.707805
x_{110}	$G^2_{\text{Li}}(\eta = 200.0, r_s = 0.25)$[H of HOH]		0.461105	1.11888	0.657777

Supplementary Table 14. Summary of training and validation data used for methane clathrate nucleation. The number of distinct configurations for which shooting results have been recorded and the number of outcomes are shown for each temperature from TPS training data and from the committor validation data.

	TPS		Committor validation	
Temperature	Configurations	Shooting results (A — B)	Configurations	Shooting results (A — B)
270 K	661	357 — 304	35	289 — 258
275 K	558	259 — 299	39	356 — 313
280 K	982	536 — 446	53	304 — 255
285 K	1197	646 — 551	33	280 — 299
all	3398	1798 — 1600	160	1229 — 1125

Supplementary Table 15. Neural network architecture used for clathrate formation. The rows show the number of units per layer.

Layer name	Number of units
Input Dimension	23
Linear1 + ELU1	23
Linear2 + ELU2	11
Linear3 + ELU3	7
Linear4 + ELU4	5
Linear5 + ELU5	4
Linear6 + ELU6	3
Linear7 + ELU7	3
Linear8 + ELU8	2
Log predictor	1

Supplementary Table 16. Description of all low-resolution (coarse grained) features used to describe the polymer folding system. Features are grouped by category (column 1). Columns 2-4 list their name, index in the feature vector, and description.

Category	Name	Index	Description
Global	U_{now}	0	Instantaneous internal energy of the polymer
	R_{now}	1	Radius of gyration of the polymer
	ani	12	Anisotropy of the system ($\frac{I_3}{I_1} - 1$)
	R_{perif}	13	Radius of gyration of the peripheral (non-core) particles
	Q_4	14	Global Steinhardt bond order parameter
	Q_6	15	Global Steinhardt bond order parameter
	I_1	16	First (smallest) eigenvalue of inertia tensor (only for core particles)
	I_2	17	Second eigenvalue of inertia tensor (only for core particles)
	I_3	18	Third (largest) eigenvalue of inertia tensor (only for core particles)
	$I_1^{(per)}$	19	First (smallest) eigenvalue of inertia tensor (only for peripheral particles)
	$I_2^{(per)}$	20	Second eigenvalue of inertia tensor (only for peripheral particles)
	$I_3^{(per)}$	21	Third (largest) eigenvalue of inertia tensor (only for peripheral particles)
Local environment of selected particles	$n1_1$	28	Environment of the first particle in the polymer
	$n1_n$	29	Environment of the last particle in the polymer chain
	$n1_{max}$	30	Maximum of $n1_1$ and $n1_n$
	$n1_{nhalf}$	31	Environment of a particle in middle of the polymer chain
Loops and chains	$alength$	2	Various measures to asses the collective behaviour of peripheral particles which are organized in "loops", "chains" and "fragments" "loops" are leaving the crystalline core and come back; "chains" are stretches of particles with a small number of neighbors; "fragments" are chains that are terminated by the first (last) particle of the polymer on one end and the crystaline core on the other end.
	$maxlength$	3	
	$alooplength$	4	
	$maxloop$	5	
	$frag_1$	6	
	$frag_2$	7	
	$fragsum$	8	
	$chainstdvar$	26	
	$nchains$	27	
Particle counts	$parts_3$	9	Number of particles in chains
	$n_{compactpart}$	10	Number of particles in compact part of polymer
	$n_{conpartnew}$	11	Particles with ≥ 5 neighbors and for which connections $\geq$ neighbors $- 1$
	$twelves$	32	Number of particles with ≥ 11 connections
	$consum$	33	Total number of connections in the polymer
	n_{core}	34	Particles with ≥ 5 connections and for which connections $\geq$ neighbours $- 1$
Distances	$dist1$	22	Distance of first particle to center of mass of the polymer
	$dist2$	23	Distance of last particle to center of mass of the polymer
	$enddist_{min}$	24	Minimum distance of terminal particles w.r.t. core particles
	$enddist_{max}$	25	Maximum distance of terminal particles w.r.t. core particles

Supplementary Table 17. Neural network architectures used for the polymer folding described with low-resolution features. The rows show the number of units for Linear and Resunit layers, the droput fraction for dropout layers. We used the element-wise ELU as activation function in each residual unit. The linear layers only reduced the width and used no activation function.

Layer name	Number of units / Dropout fraction
Input dimension	36
Linear1	24
Dropout1	0.2
Resunit1	$[24] \times 4$
Linear2	16
Dropout2	0.14
Resunit2	$[16] \times 4$
Linear3	11
Dropout3	0.1
Resunit3	$[11] \times 4$
Linear4	8
Dropout4	0.07
Resunit4	$[8] \times 4$
Log predictor	1

Supplementary Table 18. Neural network architectures used for the polymer folding described with high-resolution features. The rows show the number of units for Linear and Resunit layers, the droput fraction for dropout layers. We used the element-wise ELU as activation function in each residual unit. The linear layers only reduced the width and use no activation function.

Layer name	Number of units / Dropout fraction
Input dimension	384
Linear1	43
Dropout1	0.04
Resunit1	$[43] \times 4$
Linear2	5
Dropout2	0.04
Resunit2	$[5] \times 4$
Log predictor	1

Supplementary Table 19. Minimum, maximum and value ranges for high level descriptors in the polymer dataset used as inputs in the symbolic regression. The expressions in Suplementary Tables 20, 21 and 22 use the rescaled (to be $\in [0,1]$) versions of these descriptors.

Formula sign	Collective variable definition	Minimum	Maximum	Max - Min
Q_6	Global Steinhardt bond order parameter	0.0349859	0.522478	0.4874921
U_{now}	Instantaneous internal energy of the polymer	-2.35139	-0.937543	1.413847
$consum$	Total number of connections in the polymer	100	900	800
$twelves$	Number of particles with ≥ 11 connections	0	31	31
I_3	Third (largest) eigenvalue of inertia tensor (only for core particles)	0	570.964	570.964

Supplementary Table 20. Selected symbolic regression results for polymer folding using two or three of the most relevant descriptors (Q_6, U_{now}, $consum$) as inputs. Expressions use the scaled descriptors (scaled to be $\in [0,1]$) as inputs. See Supplementary Table 19 for the original ranges in the dataset. Expressions are sorted from lowest to highest validation loss (top to bottom) for each regularization value λ separately. All factors were rounded to 4 significant digits.

Input descriptors	λ	Validation loss	Frequency	Final expression
Q_6, U_{now}	0.01	0.355489	7/7	$q_B = 9.625Q_6 - 6.297U_{now}$
	0.001	0.349010	3/6	$q_B = -10.10U_{now} + 3.269\log Q_6 + 9.593$
		0.349438	1/6	$q_B = 10.312Q_6^{0.4623} - 10.20U_{now}$
		0.350524	2/6	$q_B = 7.278Q_6 - 10.08U_{now} + 3.595$
	0.0001	0.348566	2/6	$q_B = Q_6\left(11.85 - 12.25U_{now}\right) + \frac{0.5707 - 1.806U_{now}}{Q_6}$
		0.349010	3/6	$q_B = -10.10U_{now} + 3.269\log Q_6 + 9.593$
Q_6, U_{now}, $consum$	0.01	0.355489	3/7	$q_B = 9.625Q_6 - 6.297U_{now}$
		0.361429	4/7	$q_B = 15.33consum - 6.619$
	0.001	0.349080	4/5	$q_B = 5.215Q_6 + 5.318consum - 6.887U_{now}$
		0.349438	1/5	$q_B = 10.312Q_6^{0.4623} - 10.20U_{now}$
	0.0001	0.347718	1/3	$q_B = 4.681Q_6 + 3.640consum - 8.077U_{now} + 1.901 - 24.88\exp\left(-14.49Q_6\right)$
		0.347805	1/3	$q_B = 5.322Q_6 - 15.70consum\ U_{now} + 6.509\log\left(consum\right) + 7.728$
	0.00001	0.347417	1/5	$q_B = Q_6\left(13.51 - 16.02U_{now}\right) - 17.52\exp\left(-2.277Q_6 - 4.063consum\right)$
		0.347434	1/5	$q_B = 10.41Q_6 + 2.045consum - 5.126U_{now}\exp\left(0.9648Q_6\right) - 80.13\exp\left(-15.34consum\right)$

Supplementary Table 21. Selected symbolic regression results for polymer folding using the four most relevant descriptors (Q_6, U_{now}, *consum*, *twelves*) as inputs. Expressions use the scaled descriptors (scaled to be $\in [0, 1]$) as inputs. See Supplementary Table 19 for the original ranges in the dataset. Sorted from lowest to highest validation loss (top to bottom) for each regularization value λ separately. All factors were rounded to 4 significant digits.

Input descriptors	λ	Validation loss	Frequency	Final expression
Q_6, U_{now}, *consum*, *twelves*	0.01	0.355489	3/7	$q_B = 9.625Q_6 - 6.297U_{now}$
		0.361429	4/7	$q_B = 15.33consum - 6.619$
	0.001	0.349080	6/7	$q_B = 5.215Q_6 + 5.318consum - 6.887U_{now}$
		0.349438	1/7	$q_B = 10.312Q_6^{0.4623} - 10.20U_{now}$
	0.0001	0.346360	1/5	$q_B = Q_6 (10.33 - 8.513U_{now}) + 2.572twelves - 3.509U_{now} - 92.68 \exp(-15.24consum)$
		0.346813	1/5	$q_B = 2.304twelves - 7.694U_{now} + 3.363 \log(consum) - \frac{2.202consum}{Q_6} + 9.754$
		0.347029	1/5	$q_B = 1.949consum + 2.335twelves - 7.725U_{now} + 2.445 \log Q_6 + 5.912$
	0.00001	0.346518	1/4	$q_B = Q_6 (3.12consum - 10.74U_{now} + 8.375) + \frac{1.221twelves - 1.075U_{now}}{Q_6}$
		0.346971	1/4	$q_B = 4.987Q_6 - 7.888consum + 2.587twelves - 7.997U_{now} + 4.495 \log consum + 9.858$
	0.000001	0.346346	1/4	$q_B = Q_6 (10.90 - 9.437U_{now}) + 2.558twelves + 0.9114U_{now}^2 - 4.069U_{now} - 88.11 \exp(-14.93consum)$
		0.347029	3/4	$q_B = 1.949consum + 2.335twelves - 7.725U_{now} + 2.445 \log Q_6 + 5.912$

Supplementary Table 22. Selected symbolic regression results for polymer folding using the five most relevant descriptors (Q_6, U_{now}, *consum*, *twelves* and I_3) as inputs. Expressions use the scaled descriptors (scaled to be $\in [0,1]$) as inputs. See Supplementary Table 19 for the original ranges in the dataset. Sorted from lowest to highest validation loss (top to bottom) for each regularization value λ separately. All factors were rounded to 4 significant digits.

Input descriptors	λ	Validation loss	Frequency	Final expression
Q_6, U_{now}, *consum*, *twelves*, I_3	0.01	0.355489	2/8	$q_B = 9.625Q_6 - 6.297U_{now}$
		0.361429	6/8	$q_B = 15.33consum - 6.619$
	0.001	0.346748	1/8	$q_B = Q_6\,(7.109 - 8.871I_3) + 6.479consum - 8.019U_{now}$
		0.346855	6/8	$q_B = 5.497Q_6 + 7.518consum - 5.110I_3 - 7.416U_{now}$
		0.349438	1/8	$q_B = 10.312Q_6^{0.4623} - 10.20U_{now}$
	0.0001	0.345614	1/6	$q_B = 5.090Q_6 - 4.568I_3 + 2.042twelves - 7.380U_{now} + 2.554\log(consum) + 5.076$
		0.346221	2/6	$q_B = 5.194Q_6 + 6.238consum - 4.967I_3 + 1.832twelves - 7.003U_{now}$
		0.346237	2/6	$q_B = 6.905consum - 4.725I_3 - 7.711U_{now} + 2.604\log Q_6 + 5.030$
		0.346855	1/6	$q_B = 5.497Q_6 + 7.518consum - 5.110I_3 - 7.416U_{now}$
	0.00001	0.346855	1/5	$q_B = 5.497Q_6 + 7.518consum - 5.110I_3 - 7.416U_{now}$
	0.0000001	0.345963	2/6	$q_B = 5.297Q_6 + 7.221consum - 4.845I_3 - 7.074U_{now} - 3.228\exp(-30.65twelves)$
		0.346221	2/6	$q_B = 5.194Q_6 + 6.238consum - 4.967I_3 + 1.832twelves - 7.003U_{now}$
		0.346237	1/6	$q_B = 6.905consum - 4.725I_3 - 7.711U_{now} + 2.604\log Q_6 + 5.030$

Supplementary Table 23. Neural network architecture used for Mga2 assembly. The rows show the number of units for Resunit layers, the droput fraction for dropout layers. All residual units used the element-wise ELU activation function.

Layer name	Number of units
Input Dimension	36
Alpha dropout0	0.1
Linear1 + SELU1	36
Linear2 + SELU2	14
Linear3 + SELU3	6
Resunit1	$[6] \times 4$
Resunit2	$[6] \times 4$
Resunit3	$[6] \times 4$
Resunit4	$[6] \times 4$
Resunit5	$[6] \times 4$
Resunit6	$[6] \times 4$
Log predictor	1

1. Arjun, Berendsen, T. A. & Bolhuis, P. G. Unbiased atomistic insight in the competing nucleation mechanisms of methane hydrates. *Proc. Natl. Acad. Sci. USA* **116**, 19305–19310 (2019).
2. Barnes, B. C., Beckham, G. T., Wu, D. T. & Sum, A. K. Two-component order parameter for quantifying clathrate hydrate nucleation and growth. *J. Chem. Phys.* **140**, 164506 (2014).
3. Rodger, P. M., Forester, T. R. & Smith, W. Simulations of the methane hydrate/methane gas interface near hydrate forming conditions conditions. *Fluid Ph. Equilibria* **116**, 326–332 (1996).
